# A pan-cancer analysis of the oncogenic role of ribonucleotide reductase subunit M2 in human tumors

**DOI:** 10.7717/peerj.14432

**Published:** 2022-11-28

**Authors:** Yaqun Li, Wenhuan Fu, Zikai Geng, Yun Song, Xionggang Yang, Tianye He, Jian Wu, Bin Wang

**Affiliations:** 1 Department of Pharmacy, Huashan Hospital, Fudan University, Shanghai, China; 2 Pharmacy School, Binzhou Medical University, Shandong, China; 3 Department of Orthopedic Surgery, Huashan Hospital, Fudan University, Shanghai, China

**Keywords:** RRM2, Pan-cancer, Immune infiltration, Prognosis, Tumor microenvironment, Hepatitis B

## Abstract

**Background:**

Recent studies have identified ribonucleotide reductase subunit M2 (RRM2) as a putative promoter of tumors. However, no systematic analysis of its carcinogenicity has been conducted.

**Methods:**

The potential functions of RRM2 in various tumor types were investigated using data from the Genotype-Tissue Expression (GTEx), the Clinical Proteomic Tumor Analysis Consortium (CPTAC), the Cancer Genome Atlas (TCGA), the Human Protein Atlas (HPA), cBioPortal, GEPIA, String, and Gene Set Enrichment Analysis (GSEA). We analyzed the difference in mRNA and protein expression, pathological stage, survival, mutation, tumor microenvironment (TME), and immune cell infiltration in relation to RRM2. Meanwhile, using TCGA and the Tumor Immune Estimation Resource 2 (TIMER 2), the associations between RRM2 expression, immune infiltration, and immune-related genes were assessed. Additionally, CCK-8, Edu and RT-PCR assays were used to validate that RRM2 acts as an oncogene in liver cancer cells and its association with HBx. A cohort of liver hepatocellular carcinoma (LIHC) patients (n=154) from Huashan Hospital was analyzed for the expression of RRM2 and the association between RRM2 and immune infiltration.

**Results:**

Using the GTEx and TCGA databases, we discovered that 28 tumors expressed RRM2 at significantly higher levels than the corresponding normal tissues. Increased RRM2 expression may be predictive of a poor overall survival (OS) in patients with seven different cancers. GO, KEGG, and GSEA analyses revealed that the biological process of RRM2 was associated with the regulation of carcinogenic processes and immune pathways in a variety of tumor types. The expression of RRM2 was highly correlated with maker genes involved in immune activation and immunosuppression, immune checkpoints, DNA mismatch repair system (MMR), and the infiltration levels of Tregs and macrophages (TAMs), suggesting that the carcinogenic effect of RRM2 may be achieved by regulating immune related genes. Moreover, as demonstrated by CCK-8 and Edu assays, RRM2 was an oncogene in liver cancer cells. We confirmed for the first time that RRM2 was significantly upregulated by HBx, suggesting that RRM2 may be a key regulator of LIHC induced by HBV. IHC analysis validated the upregulated expression of RRM2 protein and its correlation with immune infiltration makers in a LIHC patient cohort.

**Conclusion:**

RRM2 may be a valuable molecular biomarker for predicting prognosis and immunotherapeutic efficacy in pan-cancer, particularly in LIHC.

## Introduction

The tumor immune microenvironment (TIME) is among the leading causes of malignant progression, involving a complex network of interactions between tumor cells and the immune system (Nelson et al., 2021; Cioni et al., 2020; Da Silva et al., 2021). Cancer cells can develop sophisticated strategies to evade the immune system’s attack, most notably through the use of immune checkpoint-related genes (Zeng et al., 2021). Immune checkpoint-related genes may be involved in immunosuppressive mechanisms, posing a significant barrier to successful immunotherapy (Mo et al., 2020; Cao et al., 2020). Immune checkpoint blockade therapy, which revolutionized cancer treatment, has now become the gold standard for cancer immunotherapy (Abril-Rodriguez & Ribas, 2017). It may cause an immune system blockage, resulting in a long-term anti-cancer response (Palmieri & Carlino, 2018; Deng et al., 2021; Sheng et al., 2020; Xu et al., 2020). However, the response rate of immunotherapy patients with cancer varies, and only a small percentage of patients respond to immunotherapy, limiting its practical application (La-Beck et al., 2015). This could be due to a lack of biomarkers to direct personalized immunity toward a specific target (Hu et al., 2022). As a result, it is critical to identify potential therapeutic biomarkers for immune checkpoint inhibitors, which will aid in predicting treatment effectiveness by clearly separating responders from non-responders (Sharma et al., 2017; Friedman, Proverbs-Singh & Postow, 2016). Given that immunotherapy efficacy is related to TIME, we sought to identify oncogenes that play a critical role in TIME (Nishino et al., 2017).

RRM2, the small subunit of ribonucleotide reductase, has been identified as a tumor promoter (Zhan et al., 2021). Previous studies demonstrated a strong association between RRM2 expression level and cancer risk. RRM2 overexpression has been reported in patients with LIHC and may function as an endogenous ferroptosis controller (Mah et al., 2015). RRM2 overexpression in multiple myeloma patients’ bone marrow mononuclear cells is highly associated with international staging system (ISS) staging, bone destruction, and extramedullary infiltration (Liu et al., 2020). RRM2 overexpression has also been implicated in oral squamous cell carcinoma, where it was associated with an advanced pathological grade and a poor prognosis for patients (Wang et al., 2021). RRM2 overexpression may be predictive of endometriosis malignancy (Yang et al., 2021). Despite these trends, further investigation is required to elucidate the additional roles of RRM2 in tumor progression and development, specifically its molecular contributions to tumor immunomodulation.

Given the variable and disease-specific nature of the molecular role, it is essential to investigate the profile of an oncogene in cases of generalized carcinoma (Majumder et al., 2021). So far, no systematic analysis of the carcinogenicity of RRM2 has been conducted. In this pan-cancer study, multiple databases were used to investigate the expression patterns of RRM2 and to visualize its prognostic value landscape. We identified the genes and signaling pathways regulated by RRM2 during cancer development. Then, we explored the connection between RRM2 expression and TIME, particularly immune checkpoints and tumor-infiltrating immune cells (TIICs).

Notably, the hepatitis B pathway was identified as a KEGG significant enrichment pathway of RRM2 binding and interacting genes, indicating that RRM2 plays an important role in the occurrence and progression of cancer, particularly LIHC. Therefore, HBV-related liver cancer was selected to verify the biological function of RRM2. Combined, these results suggest that RRM2 may serve as a predictor of both cancer clinical outcomes and immunotherapy among a variety of cancers. and it may act as a carcinogen by modulating the tumor immune microenvironment, which justifies targeting RRM2 as a novel therapeutic approach.

## Materials & Methods

### Gene and protein expression analysis of RRM2 in pan-cancer

The UCSC Xena platform was used to retrieve RNA-seq data and clinical information for the TCGA transcriptome (Goldman et al., 2020). Using log2 (TPM), the expression data were transformed to eliminate duplicate and missing values (Vivian et al., 2017). In the Single Gene Analysis module of GEPIA2, the correlation between differential RRM2 mRNA expression analysis and pathological stages analysis of TCGA tumors was determined (Tang et al., 2017). The violin plots were created using log2 (TPM (transcripts per million) + 1) transformed expression data (Huo et al., 2021). The UALCAN portal was used to analyze data from CPTAC dataset. The RRM2 total protein or phosphoprotein level was compared (Chen et al., 2019; Qiu et al., 2021). The RRM2 protein expression was validated using IHC analysis from HPA database, the results were compared to TCGA RRM2 gene expression data (Uhlén et al., 2015).

### Clinical value analysis of RRM2 in pan-cancer

To begin, Cox regression analyses were performed on TCGA datasets to determine the effect of RRM2 on pan-cancer patient survival with the R packages “survival”,“survminer” “rms” “Hmisc” and “ggplot2”. We assessed OS, progression-free survival (PFI), and disease-specific survival (DSS). We visualized the univariate Cox regression analysis using the forestplot package and created a nomogram based on the optimal multivariate Cox regression analysis. GEPIA2 was utilized to obtain OS and DFS significant level map data as well as survival plots for RRM2 across tumors (Tang et al., 2019). To determine the hypothesis, the log-rank test was utilized. Then, the R packages “pROC” and “ggplot2” were employed to generate the receiver operating characteristic (ROC) curve using the RRM2 expression profile data from the TCGA databases. The area under the curve (AUC) was computed to quantify the ability to discriminate between the tumor and normal groups (Chen et al., 2021; Xie et al., 2021a; Xie et al., 2021b; Qiu et al., 2022).

### Genetic alteration analysis of RRM2 in pan-cancer

We collected data on RRM2 alteration frequency, mutation type, mutated site information, CNA (Copy number alteration), DNA methylation alterations, and 3D structure of the protein throughout TCGA malignancies using the cBioPortal for Cancer Genomics platform (Cerami et al., 2012; Gao et al., 2013).

### RRM2-related gene enrichment analysis

For the subsequent analysis of the protein-protein interaction (PPI) network, we employed the Search Tool for the Retrieval of Interacting Genes (STRING) (Szklarczyk et al., 2019). Using Cytoscape (version 3.8.2), the PPI network was constructed, visualized, and analyzed (Otasek et al., 2019). Using GEPIA2, the top 100 RRM2-associated target genes were identified, and Pearson correlation coefficients were calculated between RRM2 and selected genes. Meanwhile, using RNA-seq data from the TCGA database, the top ten correlation results in GEPIA2 correlation analysis were verified using the adjusted Spearman’s rank correlation test.

A heatmap representation of the selected genes’ expression profiles. Then, we used the “clusterProfiler” and “org.Hs.eg.db” and “ggplot2” R packages to merge and filter the top 100 RRM2-correlated genes data in order to conduct GO and Kyoto Encyclopedia of Genes and Genomes (KEGG) pathway analysis (Yu et al., 2012; Cui et al., 2020). In addition, GSEA analysis was utilized to explore the possible biological processes involving RRM2 in pan-cancer using “clusterprofiler” R package. The sets of pathway genes were curated using data from the Molecular Signatures Database (MSigDB). Enrichment significance was defined as gene sets with *P* adjust < 0.05 and FDR < 0.25 (Subramanian et al., 2015).

### Association analysis of RRM2 expression with tumor immune microenvironment

To investigate the function of RRM2 in TIME, we determined the relationship between RRM2 and marker genes for immune activating genes, immunosuppressive genes, MMR-related genes, and immune checkpoint genes using the R packages “stat” and “ggplot2” as well as Spearman’s correlation analysis. TIMER is a tool for analyzing the relationship between RRM2 expression and immune infiltrates in all TCGA tumors (Li et al., 2020; Li et al., 2017).

### Clinical samples

From 154 patients undergoing hepatic resection at Huashan Hospital, LIHC tumor tissues and paracancerous tissues were obtained. Then, all of the samples were examined using histology. Following that, histology was used to verify all of the samples. The Huashan Hospital Human Ethics Committee approved the experimental protocols, and all patients sign an informed consent form (HIRB: 2021-783).

### Cell culture and transfection

Two LIHC cell lines (HepG2, Huh-7) and one immortalized hepatocyte cell line (LO2) were cultured in Dulbecco’s Modified Eagle’s medium (DMEM/F12, BI) supplemented with 10% FBS (Gibco, Carlsbad, CA, USA). These cell lines were obtained from the cell bank of the Chinese Academy of Sciences in Shanghai. All cells were grown in a humidified atmosphere incubator at 37 °C and 5% CO_2_. Small interfering RNA negative control 5′-UUCUCCGAACGUGUCACGUTT-3′ (sense), 5′-ACGUGACACGUUCGGAGAATT-3′ (antisense); hs-RRM2 si-1 5′-GGAGUGAUGUCAAGUCCAATT-3′ (sense), 5′-UUGGACUUGACAUCACUCCTT-3′ (antisense); hs-RRM2 si-2 5′-GGAGGAGAGAGUAAGAGAATT-3′(sense), 5′-UUCUCUUACUCUCUCCUCCTT-3′ (antisense) and hs-RRM2 si-3 5′-GCAAGCGAUGGCAUAGUAATT-3′ (sense), 5′-UUACUAUGCCAUCGCUUGCTT-3′(antisense) targeting the coding region of each mRNA, were transfected into HepG2 or Huh-7 cells with Lipo3000 transfection reagent according to the manufacturer’s instructions (HANBIO, China). After 48 h, transfected cells were used for experiments. To generate stable HBx-overexpression cell lines (LO2-HBx), lentiviral vectors pLenti-HBx-HA-Zeo (GeneChem, DaeJeon, South Korea) were constructed and used for corresponding cells by lentivirus-mediated transfection. Before the subsequent experiments, G418 (GeneChem, DaeJeon, South Korea) was used for 14 days to select stable transfections. The efficiency of over-expression and knockdown were validated by qRT-PCR.

### RNA extraction and qRT-PCR

Total RNA was extracted from samples using TRIzol reagent (Pufei, Shanghai, China) and reversely transcribed into cDNA using the PrimeScript RT reagent Kit (Takara Bio, China), following by PCR amplification using SYBRs Premix Ex Tap TM II (Takara Bio, Shiga, Japan) in a real-time PCR system (Bio-Rad, Hercules, CA) (Xie et al., 2021a; Xie et al., 2021b). GraphPad Prism was used to conduct statistical analyses. The primers sequences as below: RRM2 forward-CATTGTGAGGTACAGGCGGAAG, reverse-GAAATGGTCTGAGCTGGCAGAAG, HBx forward-CCCGTCTGTGCCTTCTCATC, reverse-CCCAACTCCTCCCAGTCTTTA, ACTB forward-TGGCACCCAGCACAATGAA, reverse-CTAAGTCATAGTCCGCCTAGAAGCA. GAPDH forward-GACATCAAGAAGGTGGTGAAGCAG,reverse-GTCAAAGGTGGAGGAGTGGGT.

### Cell viability and cell growth assay

The CCK-8 and the 5-ethynyl-20-deoxyuridine (Edu) assay were used to determine cell viability and growth. According to manufacturer’s protocol, HepG2 and Huh-7 cells were seeded at a density of 2000 cells/well in 96-well plates and treated with negative control and si-RRM2. Cell viability of transfected cells at 48 h was detected using CCK-8 assay (Dojindo, Rockville, MD, USA). Furthermore, the cells were incubated with Edu (Ribobio, Guangzhao, China). Data were obtained from three separate experiments.

### Statistical analysis

R language version 3.6.2 was utilized for statistical analysis. This study displayed all data as means ± standard deviation. Student’s *t* test and GraphPad Prism 6 were applied to determine the significance of the differences.

## Results

### RRM2 is upregulated in pan-cancer

**Figure 1:**
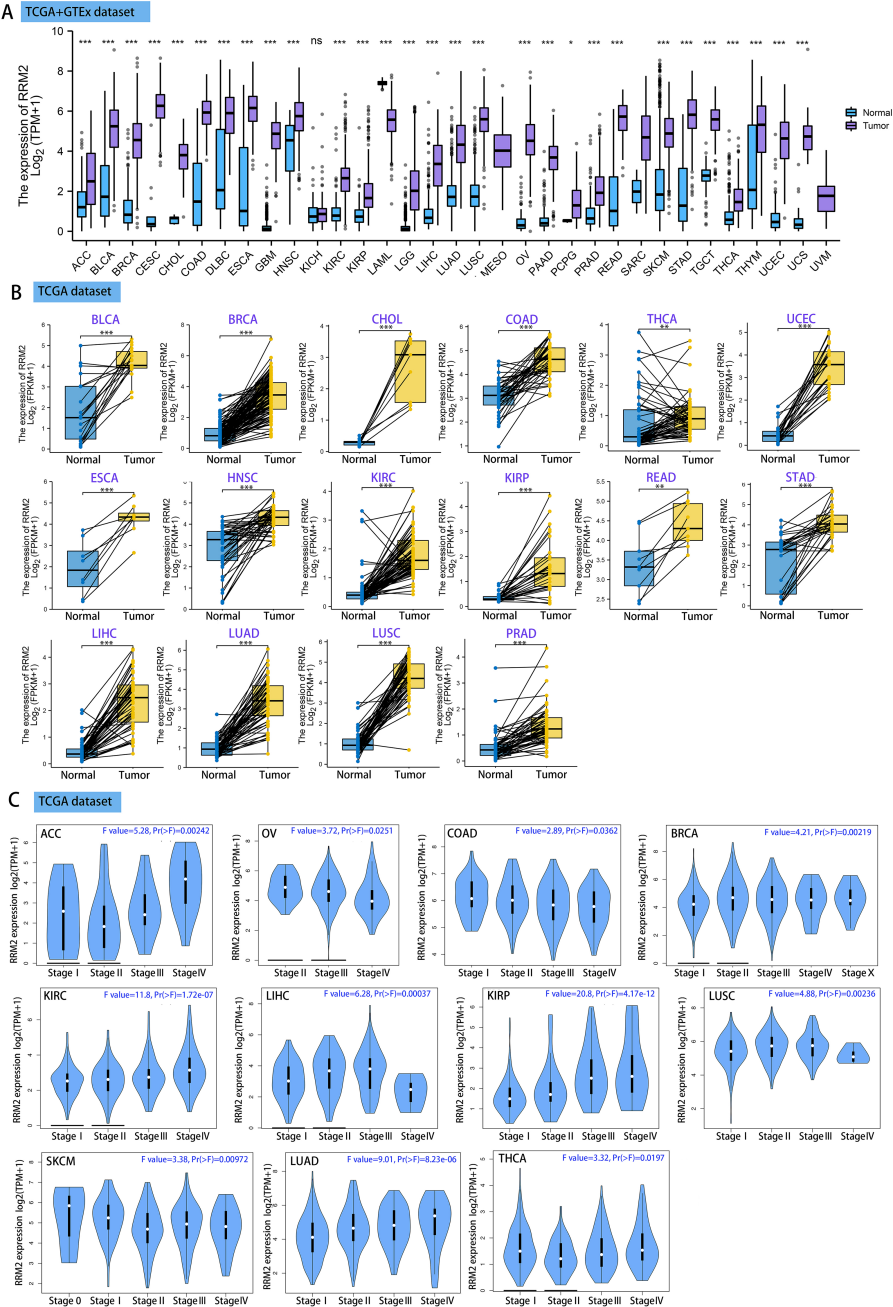
RRM2 expression profiles in normal tissues and cancers. (A) Differential RRM2 mRNA expression between TCGA cancers and GTEX normal tissues. Normal group was normal tissue in TCGA and GTEX database. (B) Pan-cancer differential expression of RRM2 in paired tumor and adjacent normal tissues in indicated tumor types from TCGA database. (C) RRM2 expression levels were assessed by the main pathological stages of ACC, COAD, BRCA, KICH, KIRC, KIRP, LIHC, LUAD, LUSC, OV, SKCM and THCA. The log2 (TPM + 1) for log-scale was used. *P < 0.05, **P < 0.01, ***P < 0.001, ****P < 0.0001.

Using GEPIA2, we analyzed the mRNA expression status of RRM2 across numerous cancer types. RRM2 expression was found to be elevated in the majority of human tumors, according to the findings (Fig. S1). RNA-seq data from the TCGA and GTex databases were used to further confirm the expression of RRM2 in pan-cancer. As depicted in Fig. 1A, RRM2 expression significantly overexpressed among 28 types of cancer. In contrast, it showed little expression in acute myeloid leukemia (LAML). In paired tumor and normal tissues from the TCGA pan-cancer database, RRM2 was highly expressed in bladder urothelial carcinoma (BLCA), breast invasive carcinoma (BRCA), cholangiocarcinoma (CHOL), colon adenocarcinoma (COAD), thyroid carcinoma (THCA), uterine corpus (UCEC), esophageal carcinoma (ESCA), head and neck cancer (HNSC), kidney renal papillary cell carcinoma (KIRP), rectum adenocarcinoma (READ), stomach adenocarcinoma (STAD), LIHC, lung adenocarcinoma (LUAD), lung squamous cell carcinoma (LUSC) and prostate adenocarcinoma (PRAD) (Fig. 1B).

**Figure 2:**
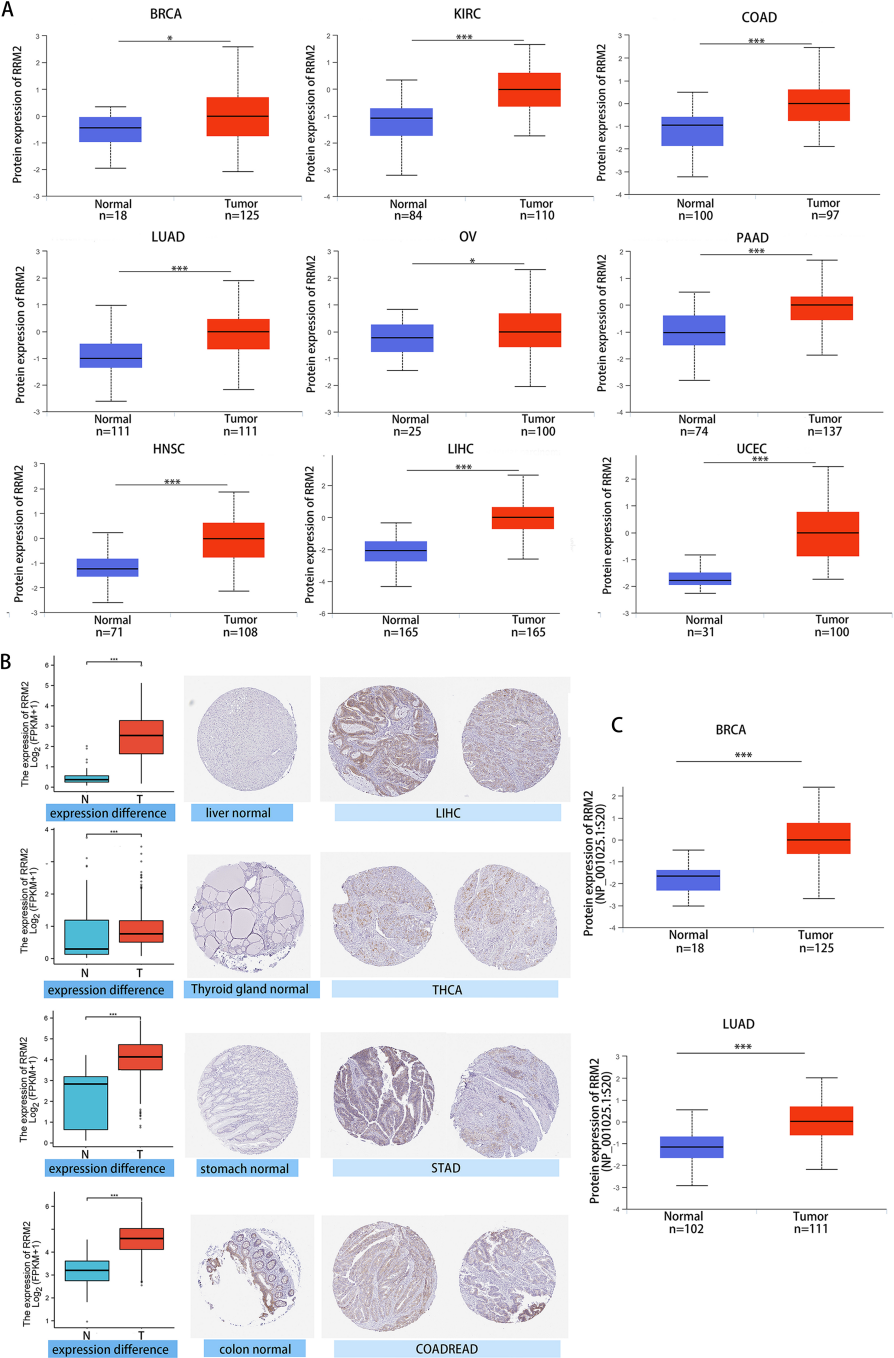
Protein level of RRM2 in human tumors. (A) Total protein level of RRM2 in normal tissue and BRCA, KIRC, COAD, HNSC, LIHC, LUAD, OV, PAAD and UCEC. Protein data was extracted and analyzed using CPTAC dataset. (B) Comparison of RRM2 gene expression (left) and immunohistochemistry images (right) in normal and tumor tissues. RRM2 protein expression was significantly higher in LIHC, THCA, STAD and COADREAD. (C) Phosphorylation analysis of RRM2 in different tumors using CPTAC dataset. Box plot representation of RRM2 phosphoprotein levels in BRCA and LUAD. *P < 0 .05, **P < 0 .01, ***P < 0.001, ****P < 0 .0001.

We investigated the relationship between RRM2 expression and pathological stages of cancer. Remarkably, we discovered clear associations in the majority of instances, including adrenocortical carcinoma (ACC), BRCA, COAD, kidney chromophobe (KICH), skin cutaneous melanoma (SKCM), KIRP, LUAD, kidney renal clear cell carcinoma (KIRC), LUSC, ovarian serous (OV), LIHC and THCA (Fig. 1C, all *P* < 0.05). In addition to evaluating RRM2 at the transcriptional level, we evaluated it at the protein level using the National Cancer Institute’s CPTAC dataset, which contains large-scale proteome data. The total protein expression of RRM2 was strongly elevated in BRCA, KIRC, COAD, HNSC, LIHC, LUAD, OV, pancreatic adenocarcinoma (PAAD), and UCEC patients (Fig. 2A, *P* < 0.05, *P* < 0.001). Meanwhile, we examined IHC information from the HPA database to validate RRM2 protein expression in CPTAC dataset and compared the results to TCGA data on RRM2 gene expression. The outcomes of the analyses of data from the HAP and TCGA database were congruent. Normal liver, thyroid gland, stomach, and colon were negative for RRM2 immunohistochemical staining, whereas tumor tissues were moderately stained (Fig. 2B). Regarding the protein phosphoprotein of RRM2, we analyzed three types of pan-cancer tumors in greater detail using the CPTAC dataset. Figure 2C summarizes RRM2 phosphorylation sites and significant differences. The S20 locus of RRM2 is more highly phosphorylated in BRCA than in normal tissues, but less so in LUAD.

### Multifaceted clinical value analysis of RRM2 in Cancers

**Figure 3:**
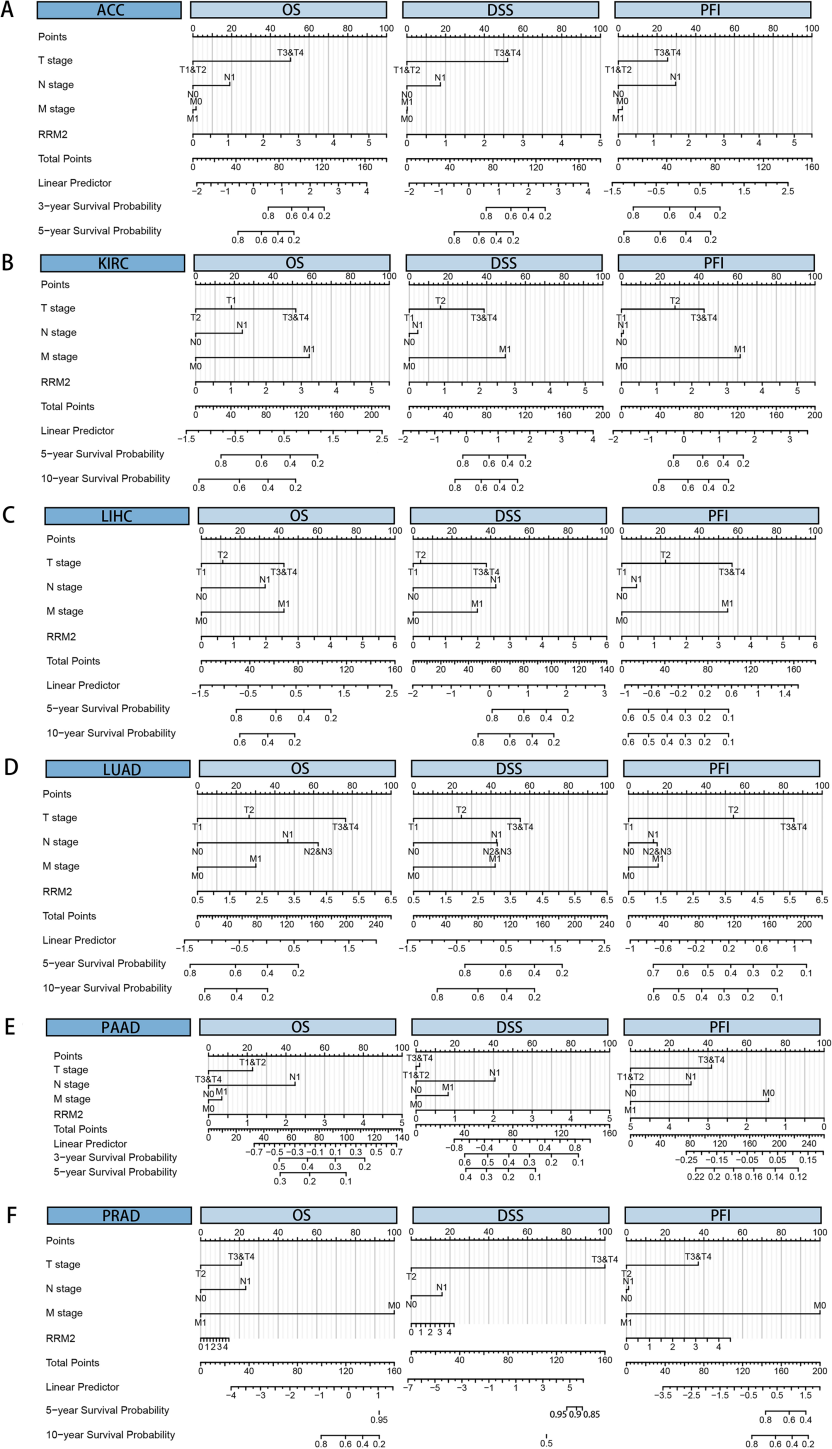
Cox regression analysis of RRM2 based on a TCGA dataset in pan-cancer. Nomogram for predicting the probability of 3-, 5- and 10-year OS , DSS, PFI in patients with ACC (A), KIRC (B), LIHC (C), LUAD (D), PAAD (E) and PRAD (F).

**Figure 4:**
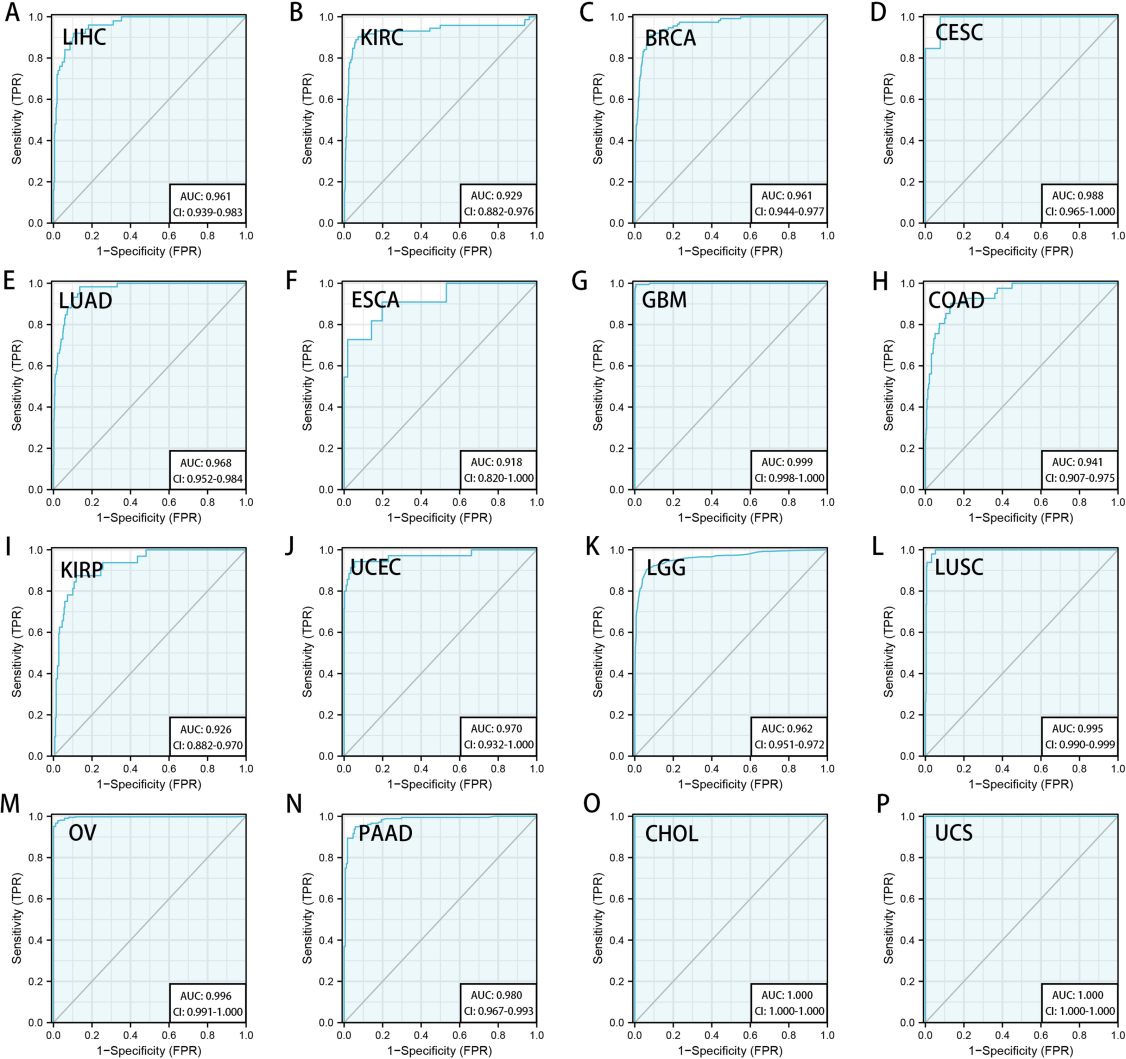
Diagnostic accuracy of RRM2 in pan-cancer. The ROC curve of RRM2 in LIHC (A), KIRC (B), BRCA (C), CESC (D), LUAD (E), ESCA (F), GBM (G), COAD (H), KIRP (I), UCEC (J), LGG (K), LUSC (L), OV (M), PAAD (N), CHOL (O), UCS (P). AUC: area under the curve. ROC: receiver operating characteristic.

We downloaded the TCGA mRNA sequencing and clinical data of 33 cancer types from the UCSC Xena platform and used the Cox proportional hazards model to examine the prognostic assessment value of RRM2 in pan-cancer (Table S1). Figure S2 shows the forest plots indicating RRM2 as a high-risk factor for KIRP, KIRC, ACC, LIHC, LUAD, PAAD, brain lower grade glioma (LGG) and PRAD of OS prognosis assessment in pan-cancer, and RRM2 expression was clearly correlated with worse DSS in LIHC, ACC, BLCA, PAAD, PRAD, KIRP, LGG, LUAD, pheochromocytoma and paraganglioma (PCPG) and KIRC. A nomogram integrating RRM2 expression and pathological stage, an independent clinical risk factor, was developed (Fig. 3). The C-index in ACC, KIRC, and KICH indicated the prediction results match the observation results perfectly, as shown in Table S2. Using the Mantel-Cox test and the GEPIA2 database, we analyzed the survival contribution of RRM2 in various cancer types; the survival maps supplemented by OS and disease-free survival (DFS) curves are depicted in Fig. S3. High RRM2 transcription levels were associated with a poor OS prognosis for cancers, including cervical squamous cell carcinoma and endocervical adenocarcinoma (CESC, logrank *P* = 0.029), ACC (logrank *P* = 0.00033), KIRC (logrank *P* = 0.021), KIRP (logrank *P* = 0.00028), LGG (logrank *P* = 1.8e−06), LIHC (logrank *P* = 0.00058), LUAD (logrank *P* = 1.5e−05), PAAD (logrank *P* = 0.0058). The analysis of DFS data revealed that patients in ACC (logrank *P* = 0.00042), KIRC (logrank *P* = 0.047), KIRP (logrank *P* = 0.00017), LGG (logrank *P* = 0.0014), LIHC (logrank *P* = 0.00032), LUAD (logrank *P* = 0.019), PAAD (logrank *P* = 0.00038), PRAD (logrank *P* = 0.0093), SKCM (logrank *P* = 0.027), THCA (logrank *P* = 0.0048). ROC curve analysis indicates that RRM2 in LIHC (AUC = 0.961, 95% CI [0.030–0.983]), KIRC (AUC = 0.929, 95% CI [0.882–0.976]), BRCA (AUC = 0.961, 95% CI [0.944–0.977]), CESC (AUC = 0.988, 95% CI [0.965–1.000]), LUAD (AUC = 0.968, 95% CI [0.952–0.984]), ESCA (AUC = 0.918, 95% CI [0.820–1.000]), glioblastoma multiforme (GBM, AUC = 0.999, 95% CI [0.998–1.000]), COAD (AUC = 0.941, 95% CI [0.907–0.975]), KIRP (AUC = 0.926, 95% CI [0.882–0.970]), UCEC (AUC = 0.970, 95% CI [0.932–1.000]), LGG (AUC = 0.962, 95% CI [0.951–0.972]), LUSC (AUC = 0.995, 95% CI [0.990–0.999]), OV (AUC = 0.996, 95% CI [0.991–1.000]), PAAD (AUC = 0.980, 95% CI [0.967–0.993]), CHOL (AUC = 1.000, 95%CI [1.000–1.000]), uterine carcinosarcoma (UCS, AUC = 1.000, 95% CI [1.000–1.000]) performed well in the diagnostic accuracy (Fig. 4).

### Mutation landscape of RRM2 in cancers

**Figure 5:**
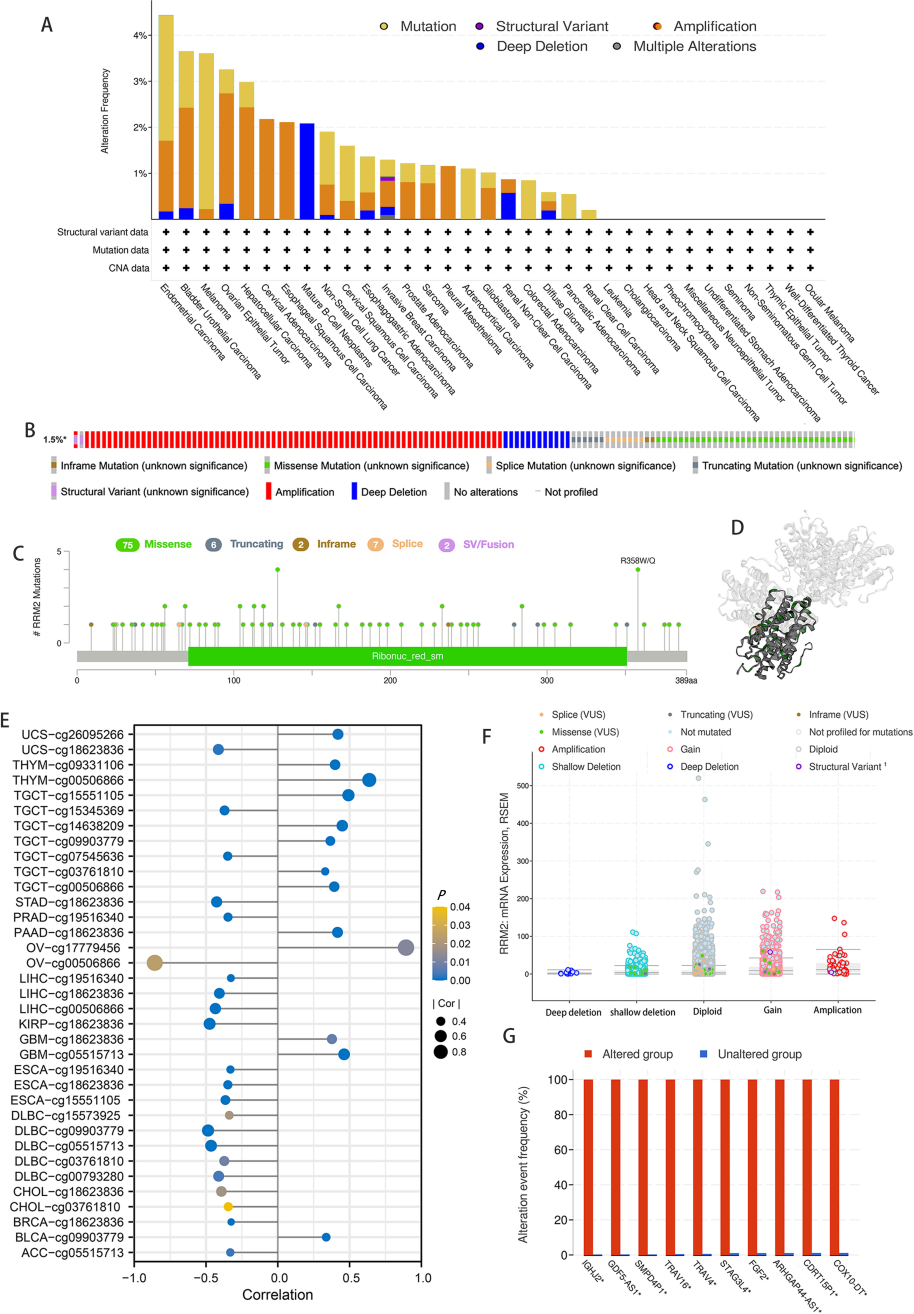
Mutation feature of RRM2 in TCGA tumors. (A) The alteration frequency with mutation type of RRM2 in TCGA pan-cancer datasets. (B) Summary of RRM2 structural variant, mutations, and copy-number alterations. (C) The mutation types, number, and sites of the RRM2 genetic alterations. (D) Location of variants on the 3D protein structure of RRM2. (E) Correlations between mRNA level of RRM2 and DNA methylation. (F) The alteration types of RRM2 in pan-cancer. (G) The related genes alteration frequency in RRM2 altered group and unaltered group.

According to the results of our analysis, RRM2 mutations include missense, inframe, splice site, truncating, amplification, and deep deletions. Melanoma patients exhibited the highest frequency of RRM2 alterations, with “mutation” being the predominant type, whereas the “amplification” type of CNA and copy number “deep deletion” were the predominant types in LIHC and Mature B-cell Neoplasms, respectively (Fig. 5A). Miss-mutation, amplification, and deep deletion are the most common types of RRM2 mutations (Fig. 5B). Figure 5C depicts RRM2 gene modification types, sites, and case counts. Between amino acids 0 and 389, we detected a total of 92 mutation sites. The RRM2 missense mutation was the most prevalent type alteration, whereas the R358W/Q mutation was identified in one case of GBM and two cases of UCEC. These mutations were dispersed throughout the entire protein sequence and 3D structure (Fig. 5D). Spearman’s correlation was utilized to examine the relationship between promoter DNA methylation and RRM2 expression levels (Fig. 5E). RRM2 expression was significantly inversely correlated with DNA methylation in patients with OV (cg00506866), diffuse large B-cell lymphoma (DLBC, cg09903779, cg00793280, cg05515713), KIRP (cg18623836), LIHC (cg00506866, cg18623836), STAD (cg18623836) and UCS (cg18623836) (−1 < Pearson *r* ≤  − 4.0). Amplification, gain function, and diploidy were the most common putative copy-number alterations of RRM2 (Fig. 5F). IGHJ2, GDF5-AS1, SMPD4P1, TRAV16, TRAV4, STAG3L4, FGF2, ARHGAP44-AS1, CDRT15P1, and COX10-DT gene alterations were more prevalent in the altered groups (Fig. 5G). These findings may merit additional in-depth investigation.

### Enrichment analysis of RRM2-related partners

**Figure 6:**
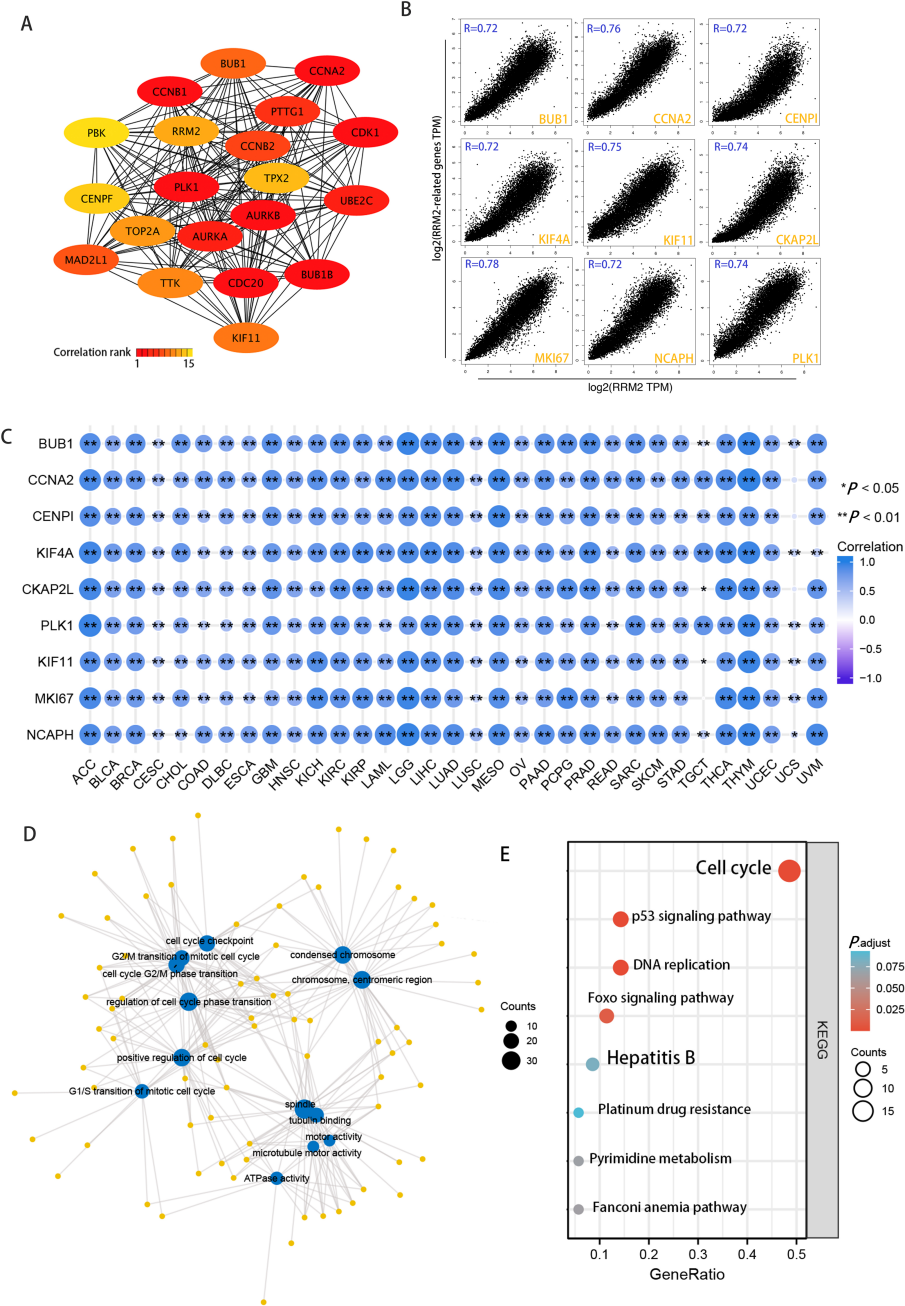
RRM2-related gene enrichment and pathway analysis. (A) STRING protein network map of experimentally determined RRM2-binding proteins. (B) Expression correlation between RRM2 and representative genes (BUB1, CCNA2, CENPI, KIF4A, CKAP2L, PLK1, KIF11, MKI67, NCAPH) of the top RRM2-correlated genes in TCGA projects as determined by GEPIA2. (C) Heatmap representation of the expression correlation data between RRM2 and BUB1, CCNA2, CENPI, KIF4A, CKAP2L, PLK1, KIF11, MKI67 and NCAPH in the TCGA tumors. (D) The regulatory network for the molecular function data in GO analysis. (E) KEGG pathway analysis based on the RRM2-binding and interacted genes.

Using the STRING tool, we obtained experimentally detected RRM2-binding proteins, and the PPI network of proteins was shown in Fig. 6A. The GEPIA2 tool was then used to identify the top 100 genes that correlate with RRM2 expression in combining TCGA pan-cancer expression. As illustrated in Fig. 6B, RRM2 expression was positively associated with that of BUB1 (BUB1 Mitotic Checkpoint Serine/Threonine Kinase, *R* = 0.72), CCNA2 (Cyclin A2, *R* = 0.76), CENPI (Centromere Protein I, *R* = 0.72), CKAP2L (Cytoskeleton Associated Protein 2 Like, *R* = 0.74), PLK1 (Polo Like Kinase 1, *R* = 0.74), KIF4A (Kinesin Family Member 4A, *R* = 0.72), KIF1 (Kinesin Family Member 1, *R* = 0.75), MKI67 (Marker of Proliferation Ki-67, *R* = 0.75), NCAPH (Non-SMC Condensin I Complex Subunit H, *R* = 0.72) genes (all *P* < 0.001). The majority of tumors exhibited a positive relationship between RRM2 and the nine genes listed above, as indicated by the results of a heat map (Fig. 6C). As shown in Fig. 6D, GO analysis determined that RRM2 was primarily involved in cell cycle checkpoint, ATPase activity, and condensed chromosome. Several pathways, including “Hepatitis B”, “p53 signaling pathway”, and “cell cycle” were identified as the KEGG pathways most significantly enriched, suggesting that RRM2 played a crucial part in the development and progression of cancers, particularly HBV-related LIHC (Fig. 6E). Meanwhile, GSEA was used to identify functional RRM2 enrichment (Fig. S4). High expression of RRM2 is implicated in numerous carcinogenic and immune-related processes, including PLK1 pathways, Forkhead box M1 (FOXM1) pathways, P53 signaling pathways, immunoregulatory interactions between a lymphoid and a nonlymphoid cell, and DNA repair and methylation.

### Correlation of RRM2 expression with tumor immune microenvironment

**Figure 7:**
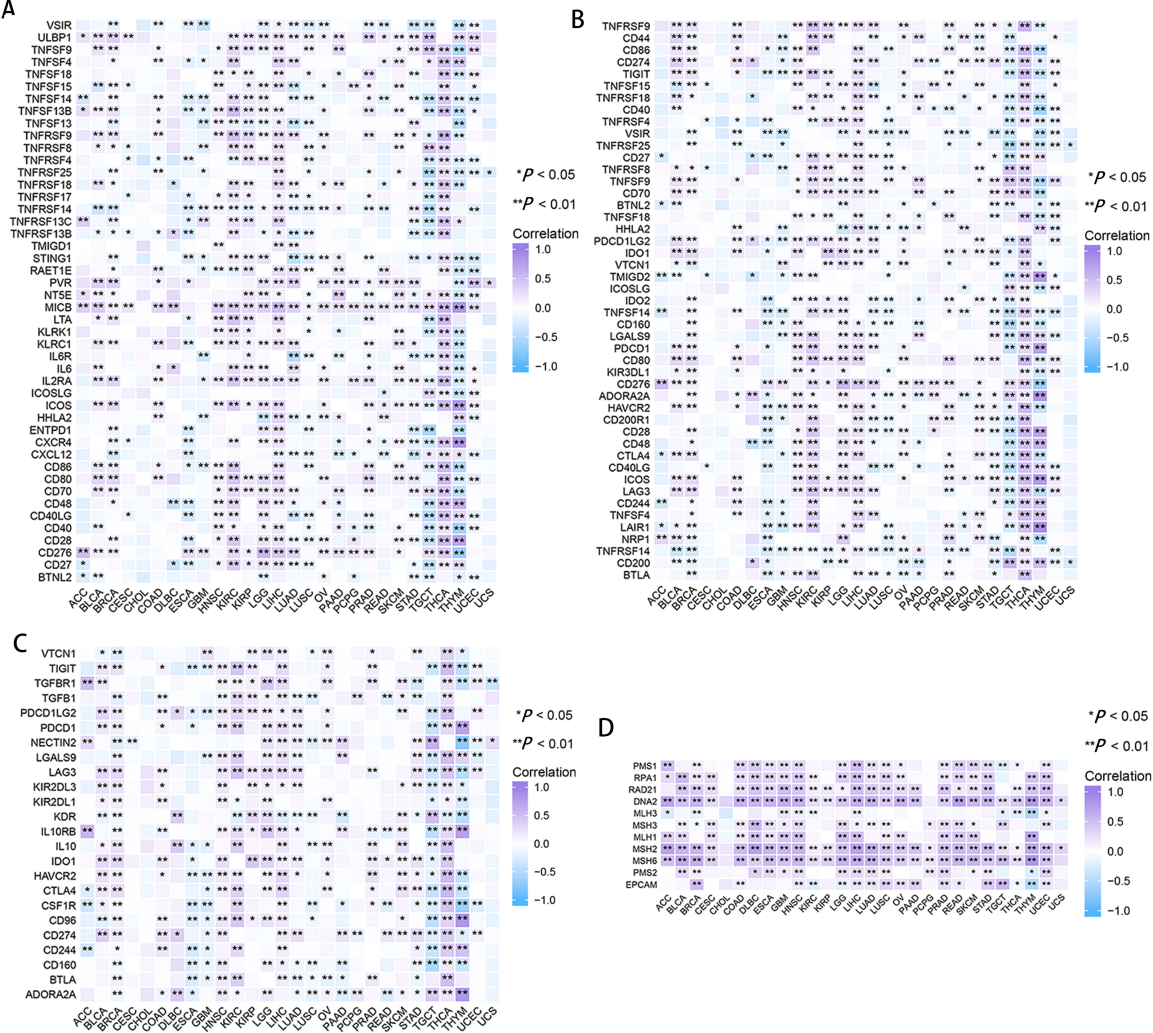
Correlation between RRM2 gene expression and tumor immune microenvironment. The heatmap represents the correlation between RRM2 expression and immunoregulation related genes, including immune activating genes (A), immune checkpoint genes (B), immunosuppressive status related genes (C), and MMR-related genes (D) using the TCGA database. *P < 0 .05, **P < 0 .01, ***P < 0.001, ****P < 0.0001.

In CESC, ESCA, GBM, LUSC, and thymoma (THYM), RRM2 had a negative correlation with marker genes of immune activating genes, such as VSTR, TNFSF14, TNFRSF14, and STING1 (Fig. 7A). RRM2 was associated with immunosuppressive genes, including PDCD1LG2, IDO1, HAVCR2 and CD274, in COAD, HNSC, DLBC, BLCA, THCA, OV, KIRP, LGG, LIHC, SKCM, and KIRC (Fig. 7C). Additionally, RRM2 expression was strongly correlated with over 30 immune checkpoint markers, including TNFRSF9, TNFSF9, CD70, LGALS9, PDCD1, CD276, HAVCR2, CD28, CD48, CTLA4, ICOS, LAG3 and LAIR, in BRCA, KIRC, LGG, LIHC, THCA, and THYM (Fig. 7B). RRM2 expression was positively correlated with the level of MMR-related genes in the majority of tumors, particularly LIHC, HNSC, GBM, LUSC, PRAD, and UCEC (Fig. 7D).

**Figure 8:**
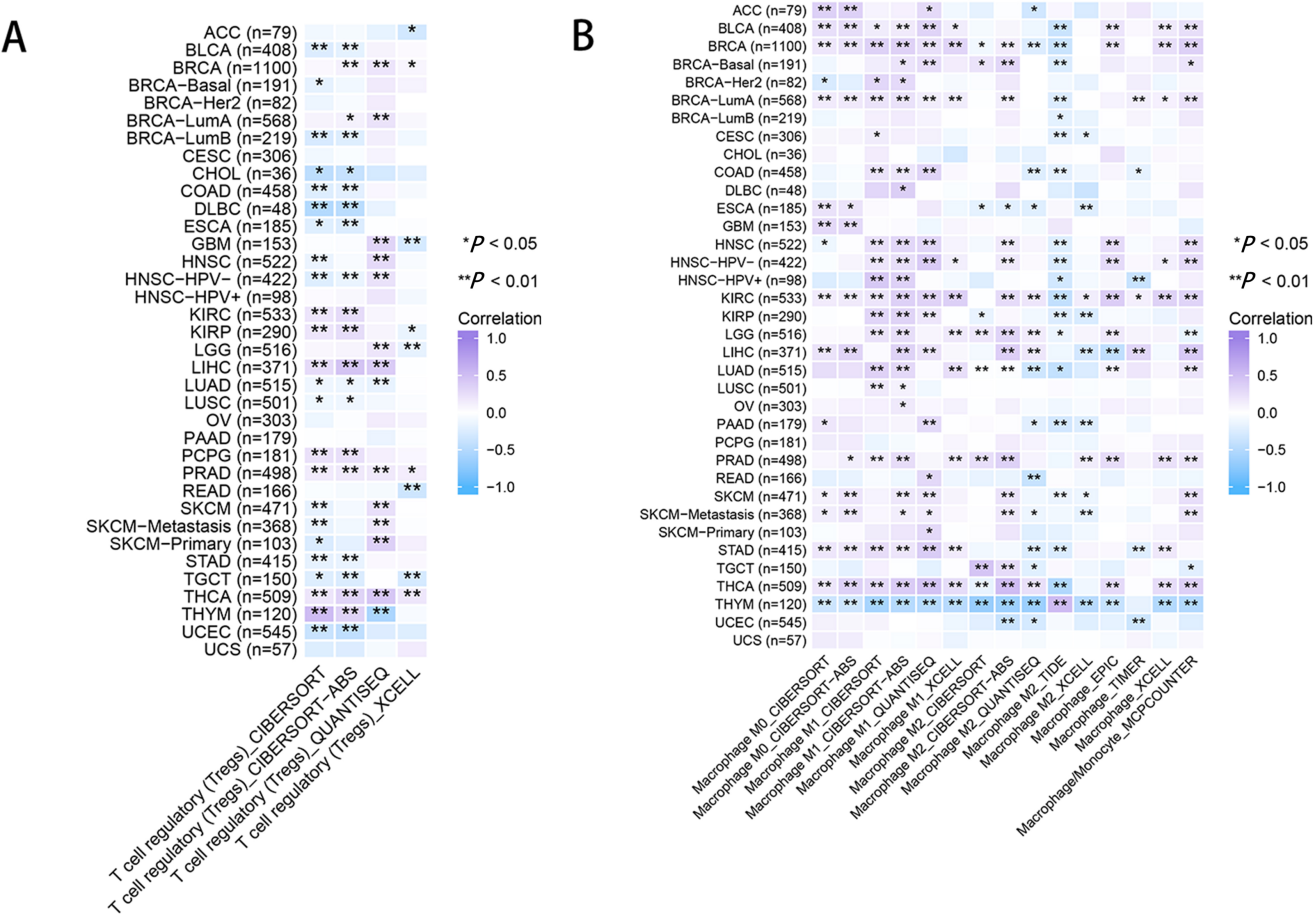
RRM2 expression correlates with the immune infiltrates of tumors. The heatmap represents the correlation between RRM2 expression level and immune infiltration of Tregs (A) and macrophages (B) using TIMER2 database. *P < 0 .05, **P < 0 .01, ***P < 0.001, ****P < 0.0001.

As for immune infiltration analysis, RRM2 expression level was linked with the estimated infiltration value of Tregs for PRAD, BRCA, LIHC, and THCA (Fig. 8A). In BLCA, COAD, ESCA, KIRP, PAAD, and STAD, RRM2 expression correlated positively with M1-like macrophages and negatively with M2-like macrophages (Fig. 8B). RRM2 expression was significantly correlated with the immune-associated cells T cells in four cancer types, cytotoxic cells in four cancer types, B cells in two cancer types, CD8+T cells in three cancer types, Treg cells in four cancer types, dendritic cells in one cancer type, macrophages in three cancer types, NK cells in six cancer types, and neutrophils in three cancer types. In particular, we discovered a significant correlation between the RRM2 expression level and seven types of immune-associated cells in THYM (Fig. S5).

### The validation of RRM2 expression and its oncogenicity

**Figure 9:**
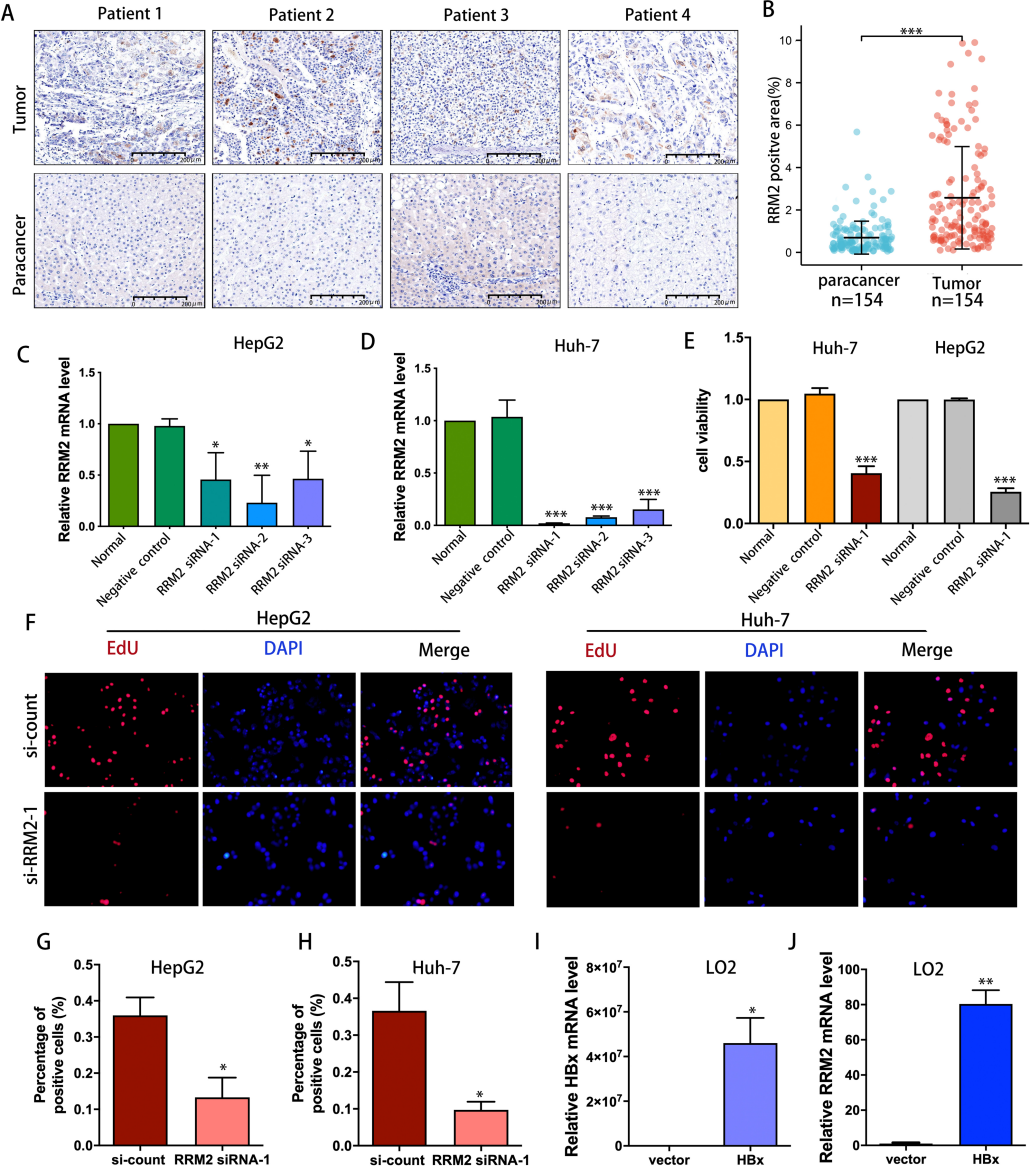
Validation of the oncogenic role of RRM2 in LIHC. (A, B) Comparison of RRM2 expression level between tumor and adjacent normal tissues in LIHC cohort (*n* = 154). Scale bar: 200 µm. Non-targeting (negative control) or siRNA targeting RRM2, as indicated. After transfection, RNA samples were then collected and relative expression ratios of RRM2 in HepG2 cells (C) and Huh-7 cells (D) were determined using qPCR analysis (*n* = 3). (E) CCK-8 was used at 48 h post-transfection to evaluate the viability of Huh-7 and HepG2 cells (*n* = 3). (F) The cell proliferation was examined by Edu incorporation assay. Scale bar: 100 µm. (G, H) Quantification of positive cells in the EdU assay (*n* = 3). The percentage of positive cells was determined by counting 1,000 cells/sample. the HA-HBx construct or empty vector was transfected into LO2 cells for 24 h. PCR analysis of HBx (I) and RRM2 (J) mRNA expression in LO2 cells with above treatment (*n* = 3). The differences between two groups were estimated by the Student’s *t* test. All values are the mean ± SD. *P < 0 .05, **P < 0 .01, ***P < 0.001, ****P < 0 .0001.

IHC was used to confirm the expression of the RRM2 protein in LIHC patient cohort from Huashan Hospital. We verified the upregulated protein expression of RRM2 in tumor tissue (*n* = 154) compared to adjacent normal tissue by IHC staining (Figs. 9A and 9B). To demonstrate the oncogene function of RRM2, we conducted experiments with liver cancer cells. We successfully suppressed RRM2 expression in HepG2 and Huh-7 cells using three RRM2 siRNA (Figs. 9C and 9D). Based on the efficiency of knockout, RRM2 siRNA-1 was chosen for subsequent experiments. CCK-8 (Fig. 9E) and Edu assays (Figs. 9F–9H) showed that RRM2 knockdown significantly inhibited cell proliferation in HepG2 and Huh-7, confirming our conclusion. In addition, HBx plasmid was transfected into LO2 cells to explore the regulatory relationship of HBx on RRM2 (Fig. 9I). We found that RRM2 mRNA expression were significantly upregulated in LO2-HBx cells (Fig. 9J).

### Validating the associations of RRM2 with immunological makers of Tregs in LIHC patient cohort

**Figure 10:**
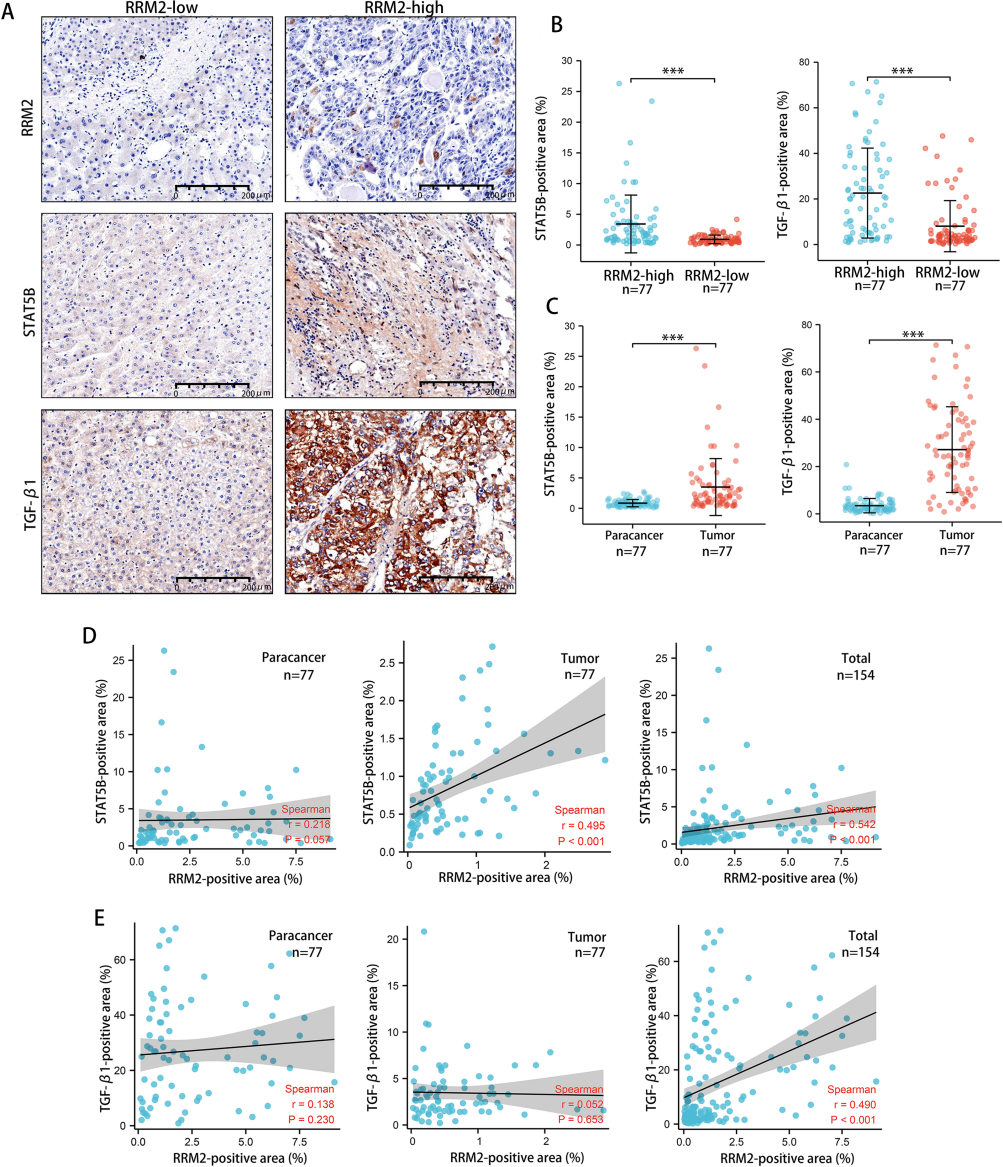
Revalidation of the relationship between RRM2 and immunological makers of Tregs (TGF- *β*1 and STAT5B) in LIHC tissues. (A, B) Comparison of the positive area of STAT5B and TGF- *β*1 between the high and low RRM2 groups in the liver cohort. (C) Comparison of the positive area of STAT5B and TGF- *β*1 between paracancerous and liver tumor tissues. (D) The correlation between STAT5B and RRM2 positive area in paracancerous and liver tumor tissues, respectively, and the total correlation in liver and paracancerous tissues. (E) The correlation between TGF- *β*1 and RRM2 positive area in paracancerous and liver tumor tissues, respectively, and the total correlation in liver and paracancerous tissues. The differences between two groups were estimated by the Student’s *t* test.The Pearson correlation coefficient was used to evaluate the correlation. All values are the mean ± SD. *P < 0 .05, **P < 0.01, ***P < 0 .001, ****P < 0 .0001.

Tumors tended to have a high abundance of Tregs cell infiltration, which was associated with protumor immunity. IHC was used to examine the expression of transforming growth factor *β*1 (TGF-*β*1) and activator of transcription (STAT)-5B protein from patients tissues. We demonstrated that both TGF-*β*1 and STAT5B expression was higher in the RRM2-high group than that in the RRM2-low group (Figs. 10A and 10B). TGF-*β*1 was significantly more strongly positive in LIHC tissues than in adjacent paracancerous tissues. STAT5B expression followed a similar pattern (Fig. 10C). A significant correlation between STAT5B and RRM2 protein levels was observed in LIHC and paracanerous tissues, as well as mixed tissues from LIHC patients (Fig. 10D). A positive correlation between TGF-*β*1 and RRM2 was also observed in mixed tissues from LIHC patients (Fig. 10E). All these findings suggested that RRM2 may play a significant role in immune infiltration in LIHC, at least in part.

## Discussion

A pan-cancer analysis with a comprehensive understanding of human cancer was typically used to identify recurring characteristics and heterogeneity during vital biological processes that contribute to a dysregulated tumor microenvironment, which is essential for identifying specific targets or characteristics for more precise and tailored treatments (Ye et al., 2019; Barger et al., 2019). This is the first pan-cancer analysis focusing on the significance of RRM2, to our knowledge. Pan-cancer analysis is of clinical value for identifying the similarities, and differences of RRM2 in various tumors. Specifically, the different results of survival analysis and the association with immune-related genes could serve as a guide and theoretical foundation for future clinical treatment, particularly tumor immunotherapy. We analyzed the expression profiles of RRM2 and its association with prognosis in various cancers. Our data revealed that RRM2 expression was significantly up-regulated compared to corresponding noncancerous tissues in a variety of cancers, regardless of gene or protein expression levels, indicating the extensive oncogenic properties of RRM2 in cancers and promising prospects for cancer research. Since phosphorylation-dephosphorylation cascade is recognized as a crucial step in oncogenesis. Consequently, we then analyzed the phosphorylation level of RRM2. In comparison to normal tissues, the RRM2 S20 phosphorylation level was significantly altered in BRCA and LUAD. The clinical significance of these posttranslational modification sites remains unknown.

Cox proportional hazards model and Kaplan–Meier analysis were used to comprehensively evaluate the association between RRM2 expression and survival time. The results of univariate and multivariate Cox regression analyses suggested that increased RRM2 expression may be associated with shorter OS, DSS, and PFI among KIRP, ACC, KIRC, PAAD, LGG, LUAD, LIHC and PRAD. Kaplan–Meier survival curves of prognostic value analysis revealed that high RRM2 expression predicted poorer OS in ACC, KIRC, KIRP, LGG, LIHC, LUAD, and PAAD, indicating that RRM2 is a risk factor in these cancers. Notably, these results identified RRM2 as a carcinogenic marker for ACC, LGG, KIRP, LIHC, KIRC, PAAD, and LUAD prognosis, regardless of the prognostic algorithm. In addition, ROC curve analysis revealed that RRM2 performed well in the diagnosis of as many as sixteen different types of tumors, especially in CHOL (AUC = 1, 95% CI [1–1]) and UCS (AUC = 1, 95% CI [1–1]). These findings suggest that high RRM2 expression is primarily responsible for carcinogenesis in the majority of tumor types, adding fresh insight to the primary pathogenesis of various tumors and providing an innovative clinical biomarker that can predict the overall survival of cancer patients.

Cancers in humans result from the accumulation of genetic mutations. Thus, we investigated RRM2 genetic mutations in human tumor samples. We discovered that mutation and amplification were the most prevalent RRM2 alterations in pan-cancer, which may trigger the onset and development of cancer. In addition, high RRM2 DNA methylation could serve as a prognostic biomarker in multiple cancers. We used pathway enrichment analyses on known RRM2-interacting proteins and RRM2-correlated genes to uncover the molecular mechanism of RRM2 in tumorigenesis and development. Results indicate that RRM2 was significantly overrepresented in immune-related pathways, specially, immunoregulatory interactions between lymphoid and non-lymphoid cell pathways are a notable immune-related pathway that modify the response of lymphoid cells to self, tumor, and pathogen antigens (Cemerski & Shaw, 2006; Nedvetzki et al., 2007). These findings indicate that RRM2 regulates the tumor immune microenvironment and ligand–receptor interactions between lymphoid and malignant tumor cells.

Next, we investigated the effect of RRM2 on TIME and evaluated whether RRM2 could be used as a biomarker of tumor cell response to immunotherapy and influence clinical outcomes *via* TIME parameters. Our initial investigation revealed that aberrantly high RRM2 expression was negatively related to immune activating maker genes in the majority of cancers, whereas it was positively correlated with immunosuppressive genes, indicating that RRM2 regulated the tumor immune microenvironment and immuno-oncological interactions. RRM2 expression was highly associated with immune checkpoint genes and MMR-related genes, indicating that RRM2 may regulate the tumor immune microenvironment to promote tumor growth. As principal components of TIME, tumor-infiltrating immune cells frequently participated in tumor behaviors such as Initiation, progression, and metastasis of cancer (Kim et al., 1991). Tumor-infiltrating immune cells are dysfunctional, unable to control tumor growth, and may even foster its advancement, resulting in immune evasion. The link between RRM2 and immune cell infiltration was investigated in this study, we observed a positive correlation between Treg infiltration levels and RRM2 in BRCA, KIRC, LIHC, PCPG, PRAD, and THCA, indicating that RRM2 contributes to Tregs exerting an inhibitory role in aiding malignant tumor cells in evading attack by cytotoxic CD8+ T cells (Shu et al., 2019; Liu, Workman & Vignali, 2016). Meanwhile, we assessed the connection between RRM2 expression and macrophage infiltration (Togashi, Shitara & Nishikawa, 2019). Infiltration of tumor-associated macrophages (TAMs) was significantly correlated with RRM2 expression in the majority of tumor types. In addition, RRM2 may directly or indirectly contribute to macrophage polarization and tumor immune evasion in BLCA, COAD, ESCA, KIRP, PAAD, and STAD (Armitage et al., 2021; Li et al., 2019). RRM2 could be an option for reducing the number of TAMs. Our finding that RRM2 played a crucial role in cancer immunology emphasized the need for future research involving larger patient cohorts to determine the clinical utility of immune checkpoint inhibitor biomarkers.

It is worth noting that KEGG pathway analysis revealed that RRM2 and HBV have a strong relationship. HBV, the leading cause of acute and chronic viral hepatitis, may contribute to the development of LIHC. In addition, the liver, as an important immune organ, provides a large and complex immune microenvironment for the development of LIHC, particularly viral hepatitis involving immune disorders, which plays a significant role in promoting the development of hepatocellular carcinoma (Xie et al., 2022; Vivier et al., 2008). Therefore, we hypothesize that RRM2 is a crucial intermediate link in the development of HBV-related LIHC. On the basis of this hypothesis, we selected LIHC as a representative tumor for future trials to validate the carcinogenicity of RRM2 and its association with HBV as well as immune infiltration. IHC analysis validated the upregulated expression of RRM2 protein and a strong positive association of RRM2 with immunological makers (TGF-*β*1 and STAT5B) of Tregs in LIHC patients cohort, which suggested that RRM2 may be a novel immune cell infiltration regulator that can control the recruitment and activation of immune cells in LIHC. HBV X protein (HBx), functioning as a viral transactivation factor, can alter multiple genes and result in the development of LIHC (Gao et al., 2021; Niu et al., 2017; Xue et al., 2020; Zhang et al., 2021). We investigated the relationship between RRM2 and HBV by analyzing the level of RRM2 expression after HBx overexpression. Notable is the fact that we confirmed for the first time that RRM2 was significantly upregulated by HBx, suggesting that RRM2 may serve as a key regulator of LIHC induced by HBV. IHC analysis also validated the upregulated expression of RRM2 protein and its correlation with immune infiltration makers in a LIHC patient cohort. Besides, we discovered that inhibiting RRM2 significantly reduced the proliferation rate of HepG2 and Huh-7 cells, which demonstrated RRM2 was an oncogene in LIHC.

Above all, RRM2 may be a valuable molecular biomarker for predicting prognosis and immunotherapeutic efficacy in pan-cancer, particularly in LIHC. We believe these findings may serve as a foundation for future functional experiments and may have eventual clinical implications. Our research has a number of limitations. Importantly, additional *in vivo* experiments and clinical trials should be conducted to validate RRM2’s role as an immune-related biomarker.

## Conclusions

Our pan-cancer analysis provides a comprehensive summary of the oncogenic roles of RRM2 in a variety of human cancers, particularly its role in LIHC and the association between RRM2 and a poor prognosis. We have shown for the first time that HBx significantly upregulates RRM2, indicating that RRM2 may be a key regulatory gene of HBV-induced LIHC. In addition, high RRM2 expression may contribute to the tumor immune microenvironment, suggesting that the carcinogenic effect of RRM2 may be realized through the regulation of immune-related genes. Consequently, RRM2 may be a useful biomarker for the prognosis and prediction of immunotherapeutic efficacy.

##  Supplemental Information

10.7717/peerj.14432/supp-1 Supplemental Information 1 Raw data exported form the Bio-Rad CFX Connect applied for data analyses and preparation for Fig 9C and D

10.7717/peerj.14432/supp-2 Supplemental Information 2 Raw data applied for data analyses and preparation for Fig 9E

10.7717/peerj.14432/supp-3 Supplemental Information 3 Raw data applied for data analyses and preparation for Fig 9F

10.7717/peerj.14432/supp-4 Supplemental Information 4 Raw data exported form the Bio-Rad CFX Connect applied for data analyses and preparation for Fig 9I

10.7717/peerj.14432/supp-5 Supplemental Information 5 Raw data exported form the Bio-Rad CFX Connect applied for data analyses and preparation for Fig 9J

10.7717/peerj.14432/supp-6 Table S1 Expression level of RRM2 in TCGA tumors vs. adjacent tissues (if available) as visualized by GEPIA2

10.7717/peerj.14432/supp-7 Table S2 The C-index of nomogram

10.7717/peerj.14432/supp-8 Figure S1 Expression level of RRM2 in TCGA tumors vs. adjacent tissues (if available) as visualized by GEPIA2

10.7717/peerj.14432/supp-9 Figure S2 The Forest plot showing OS, DSS, PFI after univariate Cox analysis in pan-cancer (A)OS, (B)DSS, (C)PFI.

10.7717/peerj.14432/supp-10 Figure S3 The survival maps and survival curves were depicted to perform OS and DFS analyses in cancers (A)OS, (B)DFS. The Kaplan-Meier curves with positive results are given.

10.7717/peerj.14432/supp-11 Figure S4 GSEA of RRM2 in pan-cancer The 10 significant pathways of RRM2 GSEA results across the indicated tumor types.

10.7717/peerj.14432/supp-12 Figure S5 Correlation analysis of immune-associated cells with RRM2 expression in pan-cancer using TCGA database

## Abbreviation

RRM2: Ribonucleotide reductase subunit M2
GTEx: The Genotype-Tissue Expression
CPTAC: The Clinical Proteomic Tumor Analysis Consortium
TCGA: The Cancer Genome Atlas
HPA: The Human Protein Atlas
GSEA: Gene Set Enrichment Analysis
TME: Tumor microenvironment
TIMER 2: Tumor Immune Estimation Resource 2
LIHC: Liver hepatocellular carcinoma
OS: Overall survival
MMR: DNA mismatch repair system
TAMs: Macrophages
TIME: Tumor immune microenvironment
ISS: International staging system
TIICs: Tumor-infiltrating immune cells
PFI: Progression-free survival
DSS: Disease-specific survival
ROC: Receiver operating characteristic
AUC: Area under the curve
CNA: Copy number alteration
PPI: Protein-protein interaction
STRING: Search Tool for the Retrieval of Interacting Genes
KEGG: Kyoto encyclopedia of genes and genomes
MSigDB: The Molecular Signatures Database
Edu: 5-ethynyl-20-deoxyuridine
LAML: Acute myeloid leukemia
BLCA: Bladder urothelial carcinoma
BRCA: Breast invasive carcinoma
CHOL: Cholangiocarcinoma
COAD: Colon adenocarcinoma
THCA: Thyroid carcinoma
UCEC: Uterine corpus
ESCA: Esophageal carcinoma
HNSC: Head and neck cancer
KIRP: Kidney renal papillary cell carcinoma
READ: Rectum adenocarcinoma
STAD: Stomach adenocarcinoma
LUAD: Lung adenocarcinoma
LUSC: Lung squamous cell carcinoma
PRAD: Prostate Adenocarcinoma
ACC: Adrenocortical carcinoma
KICH: Kidney Chromophobe
SKCM: Skin cutaneous melanoma
OV: Ovarian serous
DFS: Disease-free survival
DLBC: Diffuse large B-cell lymphoma
BUB1: BUB1 Mitotic Checkpoint Serine/Threonine Kinase
CCNA2: Cyclin A2
CENPI: Centromere Protein I
CKAP2L: Cytoskeleton Associated Protein 2 Like
PLK1: Polo Like Kinase 1
KIF4A: Kinesin Family Member 4A
KIF1: Kinesin Family Member 1
MKI67: Marker of Proliferation Ki-67
NCAPH: Non-SMC Condensin I Complex Subunit H
TAMs: TAMs tumor-associated macrophages
HBx: HBV X protein
KIRC: Kidney renal clear cell carcinoma
LGG: Brain lower grade glioma
PCPG: Pheochromocytoma and paraganglioma
CESC: Cervical squamous cell carcinoma and endocervical adenocarcinoma
PAAD: Pancreatic adenocarcinoma
GBM: Glioblastoma multiforme
UCS: Uterine carcinosarcoma
THYM: Thymoma
FOXM1: Forkhead box M1
TGF-*β*1: Transforming growth factor *β*1
STAT: Activator of transcription

## Additional Information and Declarations

### Data Availability

The following information was supplied regarding data availability: The raw measurements are available in the Supplemental File.
