# Supplementary material for: A pan-cancer analysis of the oncogenic role of ribonucleotide reductase subunit M2 in human tumors
Source: PeerJ. 2022 Nov 28;10:e14432. doi: 10.7717/peerj.14432 (PMC9744174; doi:10.7717/peerj.14432)
Supplement: Table S1 [file peerj-10-14432-s006.docx]

**Table S1. Cox proportional hazards model of RRM2 expression in pan-cancer.**

|  | OS | | | | | DSS | | | | | PFI | | | | |
| --- | --- | --- | --- | --- | --- | --- | --- | --- | --- | --- | --- | --- | --- | --- | --- |
| Cancer | N | HR (95% CI) Univariate analysis | P value Univariate analysis | HR (95% CI) Multivariate analysis | P value Multivariate analysis | N | HR (95% CI) Univariate analysis | P value Univariate analysis | HR (95% CI) Multivariate analysis | P value Multivariate analysis | N | HR (95% CI) Univariate analysis | P value Univariate analysis | HR (95% CI) Multivariate analysis | P value Multivariate analysis |
| ACC | 79 | 2.174 (1.644-2.876) | <0.001 | 2.174 (1.644-2.876) | <0.001 | 77 | 2.234 (1.651-3.021) | <0.001 | 2.234 (1.651-3.021) | <0.001 | 79 | 1.832 (1.455-2.307) | <0.001 | 1.832 (1.455-2.307) | <0.001 |
| BLCA | 413 | 1.142 (0.990-1.316) | 0.068 | 1.142 (0.990-1.316) | 0.068 | 399 | 1.226 (1.031-1.457) | 0.021 | 1.226 (1.031-1.457) | 0.021 | 414 | 1.178 (1.021-1.359) | 0.025 | 1.178 (1.021-1.359) | 0.035 |
| BRCA | 1082 | 1.085 (0.941-1.252) | 0.262 |  |  | 1062 | 1.173 (0.969-1.421) | 0.102 |  |  | 1082 | 1.168 (1.011-1.350) | 0.035 | 1.168 (1.011-1.350) | 0.035 |
| CESC | 306 | 1.129 (0.794-1.606) | 0.500 |  |  | 302 | 1.122 (0.750-1.678) | 0.577 |  |  | 306 | 0.971 (0.691-1.364) | 0.864 |  |  |
| CHOL | 36 | 1.437 (0.813-2.541) | 0.212 |  |  | 35 | 1.394 (0.763-2.548) | 0.280 |  |  | 36 | 1.237 (0.768-1.992) | 0.381 |  |  |
| COAD | 477 | 0.788 (0.590-1.053) | 0.107 |  |  | 461 | 0.727 (0.507-1.043) | 0.083 | 0.727 (0.507-1.043) | 0.083 | 477 | 0.749 (0.581-0.965) | 0.025 | 0.749 (0.581-0.965) | 0.025 |
| DLBC | 48 | 0.464 (0.199-1.079) | 0.075 | 0.464 (0.199-1.079) | 0.075 | 48 | 0.365 (0.118-1.124) | 0.079 | 0.365 (0.118-1.124) | 0.079 | 48 | 1.040 (0.473-2.287) | 0.923 |  |  |
| ESCA | 162 | 1.111 (0.801-1.540) | 0.528 |  |  | 161 | 1.116 (0.760-1.639) | 0.576 |  |  | 162 | 1.176 (0.879-1.574) | 0.275 |  |  |
| GBM | 168 | 1.203 (0.982-1.474) | 0.074 | 1.203 (0.982-1.474) | 0.074 | 155 | 1.237 (0.996-1.535) | 0.054 | 1.237 (0.996-1.535) | 0.054 | 168 | 1.073 (0.883-1.304) | 0.476 |  |  |
| HNSC | 501 | 1.023 (0.865-1.210) | 0.792 |  |  | 476 | 1.060 (0.853-1.316) | 0.600 |  |  | 501 | 1.103 (0.923-1.319) | 0.280 |  |  |
| KICH | 64 | 7.284 (3.113-17.041) | <0.001 | 7.284 (3.113-17.041) | <0.001 | 64 | 10.549 (3.749-29.679) | <0.001 | 10.549 (3.749-29.679) | <0.001 | 64 | 3.991 (2.127-7.489) | <0.001 | 3.991 (2.127-7.489) | <0.001 |
| KIRC | 539 | 1.751 (1.472-2.082) | <0.001 | 1.751 (1.472-2.082) | <0.001 | 528 | 2.232 (1.835-2.714) | <0.001 | 2.232 (1.835-2.714) | <0.001 | 537 | 1.844 (1.549-2.196) | <0.001 | 1.844 (1.549-2.196) | <0.001 |
| KIRP | 288 | 2.569 (2.019-3.270) | <0.001 | 2.569 (2.019-3.270) | <0.001 | 284 | 3.266 (2.464-4.328) | <0.001 | 3.266 (2.464-4.328) | <0.001 | 287 | 2.401 (1.939-2.972) | <0.001 | 2.401 (1.939-2.972) | <0.001 |
| LAMl | 140 | 0.966 (0.737-1.267) | 0.804 |  |  |  |  |  |  |  |  |  |  |  |  |
| LGG | 527 | 1.584 (1.392-1.803) | <0.001 | 1.584 (1.392-1.803) | <0.001 | 519 | 1.571 (1.372-1.799) | <0.001 | 1.571 (1.372-1.799) | <0.001 | 527 | 1.390 (1.244-1.554) | <0.001 | 1.390 (1.244-1.554) | <0.001 |
| LIHC | 373 | 1.387 (1.180-1.630) | <0.001 | 1.387 (1.180-1.630) | <0.001 | 365 | 1.488 (1.206-1.837) | <0.001 | 1.488 (1.206-1.837) | <0.001 | 373 | 1.286 (1.125-1.470) | <0.001 | 1.286 (1.125-1.470) | <0.001 |
| LUAD | 526 | 1.316 (1.159-1.494) | <0.001 | 1.316 (1.159-1.494) | <0.001 | 491 | 1.425 (1.214-1.674) | <0.001 | 1.425 (1.214-1.674) | <0.001 | 526 | 1.259 (1.123-1.412) | <0.001 | 1.259 (1.123-1.412) | <0.001 |
| LUSC | 496 | 0.877 (0.743-1.034) | 0.119 |  |  | 444 | 1.055 (0.810-1.373) | 0.691 |  |  | 497 | 0.975 (0.795-1.196) | 0.809 |  |  |
| MESO | 85 | 2.163 (1.623-2.883) | <0.001 | 2.163 (1.623-2.883) | <0.001 | 65 | 2.221 (1.554-3.174) | <0.001 | 2.221 (1.554-3.174) | <0.001 | 83 | 1.836 (1.349-2.501) | <0.001 | 1.836 (1.349-2.501) | <0.001 |
| OV | 377 | 0.894 (0.770-1.037) | 0.138 |  |  | 352 | 0.875 (0.746-1.027) | 0.101 |  |  | 377 | 0.939 (0.818-1.077) | 0.370 |  |  |
| PAAD | 178 | 1.655 (1.283-2.135) | <0.001 | 1.655 (1.283-2.135) | <0.001 | 172 | 1.699 (1.272-2.269) | <0.001 | 1.699 (1.272-2.269) | <0.001 | 178 | 1.692 (1.323-2.165) | <0.001 | 1.692 (1.323-2.165) | <0.001 |
| PCPG | 183 | 2.512 (0.981-6.432) | 0.055 | 2.512 (0.981-6.432) | 0.055 | 183 | 2.505 (0.829-7.568) | 0.104 |  |  | 183 | 2.410 (1.276-4.553) | 0.007 | 2.410 (1.276-4.553) | 0.007 |
| PRAD | 499 | 2.246 (1.083-4.659) | 0.030 | 2.246 (1.083-4.659) | 0.030 | 497 | 4.161 (1.517-11.413) | 0.006 | 4.161 (1.517-11.413) | 0.006 | 499 | 1.857 (1.480-2.330) | <0.001 | 1.857 (1.480-2.330) | <0.001 |
| READ | 166 | 0.665 (0.416-1.065) | 0.089 | 0.665 (0.416-1.065) | 0.089 | 160 | 1.072 (0.490-2.344) | 0.862 |  |  | 166 | 0.925 (0.590-1.451) | 0.734 |  |  |
| SARC | 263 | 1.225 (1.030-1.456) | 0.022 | 1.225 (1.030-1.456) | 0.022 | 257 | 1.229 (1.017-1.485) | 0.033 | 1.229 (1.017-1.485) | 0.033 | 263 | 1.251 (1.078-1.450) | 0.003 | 1.251 (1.078-1.450) | 0.003 |
| SKCM | 456 | 1.075 (0.925-1.249) | 0.345 |  |  | 450 | 1.088 (0.927-1.278) | 0.302 |  |  | 457 | 1.128 (0.995-1.278) | 0.061 | 1.128 (0.995-1.278) | 0.061 |
| STAD | 370 | 0.905 (0.765-1.069) | 0.240 |  |  | 349 | 0.852 (0.693-1.049) | 0.131 |  |  | 372 | 0.849 (0.713-1.012) | 0.068 | 0.849 (0.713-1.012) | 0.068 |
| TGCT | 139 | 1.547 (0.329-7.279) | 0.581 |  |  | 139 | 1.658 (0.317-8.664) | 0.549 |  |  | 139 | 1.275 (0.826-1.966) | 0.272 |  |  |
| THCA | 510 | 1.211 (0.495-2.961) | 0.675 |  |  |  |  |  |  |  | 510 | 2.349 (1.586-3.478) | <0.001 | 2.349 (1.586-3.478) | <0.001 |
| THYM | 118 | 0.608 (0.409-0.902) | 0.014 | 0.608 (0.409-0.902) | 0.014 | 118 | 0.842 (0.436-1.628) | 0.610 |  |  | 118 | 0.913 (0.688-1.213) | 0.530 |  |  |
| UCEC | 551 | 1.171 (0.970-1.415) | 0.101 |  |  | 549 | 1.177 (0.933-1.483) | 0.169 |  |  | 551 | 1.198 (1.018-1.410) | 0.030 | 1.198 (1.018-1.410) | 0.030 |
| UCS | 56 | 0.932 (0.553-1.571) | 0.792 |  |  | 54 | 0.871 (0.505-1.500) | 0.618 |  |  | 56 | 0.959 (0.589-1.561) | 0.865 |  |  |
|  |  |  |  |  |  |  |  |  |  |  |  |  |  |  |  |
| UVM | 80 | 4.050 (2.063-7.953) | <0.001 | 4.050 (2.063-7.953) | <0.001 | 80 | 4.120 (2.062-8.231) | <0.001 | 4.120 (2.062-8.231) | <0.001 | 79 | 5.177 (2.795-9.589) | <0.001 | 5.177 (2.795-9.589) | <0.001 |
