# Supplementary material for: A pan-cancer analysis of the oncogenic role of ribonucleotide reductase subunit M2 in human tumors
Source: PeerJ. 2022 Nov 28;10:e14432. doi: 10.7717/peerj.14432 (PMC9744174; doi:10.7717/peerj.14432)
Supplement: Table S2 [file peerj-10-14432-s007.docx]

**Table S2. The C-index of nomogram.**

| cancer |  | C-index |  |
| --- | --- | --- | --- |
|  | OS | DSS | PFI |
| ACC | 0.855(0.827-0.882) | 0.855(0.826-0.884) | 0.751(0.714-0.789) |
| BLCA | 0.676(0.640-0.712) | 0.738(0.702-0.775) | 0.726(0.697-0.755) |
| KIRC | 0.754(0.728-0.780) | 0.839(0.816-0.862) | 0.825(0.805-0.845) |
| LIHC | 0.712(0.678-0.745) | 0.791(0.759-0.823) | 0.663(0.641-0.685) |
| KICH | 0.981(0.970-0.992) | 0.988(0.979-0.997) | 0.875(0.801-0.949) |
| LUAD | 0.696(0.672-0.721) | 0.695(0.664-0.727) | 0.641(0.617-0.665) |
| MESO | 0.706(0.667-0.745) | 0.709(0.663-0.756) | 0.663(0.617-0.709) |
| PAAD | 0.594(0.547-0.642) | 0.621(0.571-0.672) | 0.552(0.508-0.597) |
| PRAD | 0.617(0.457-0.777) | 0.838(0.725-0.951) | 0.695(0.663-0.727) |
