# Supplementary figures and images for: A pan-cancer analysis of the oncogenic role of ribonucleotide reductase subunit M2 in human tumors
Source: PeerJ. 2022 Nov 28;10:e14432. doi: 10.7717/peerj.14432 (PMC9744174; doi:10.7717/peerj.14432)

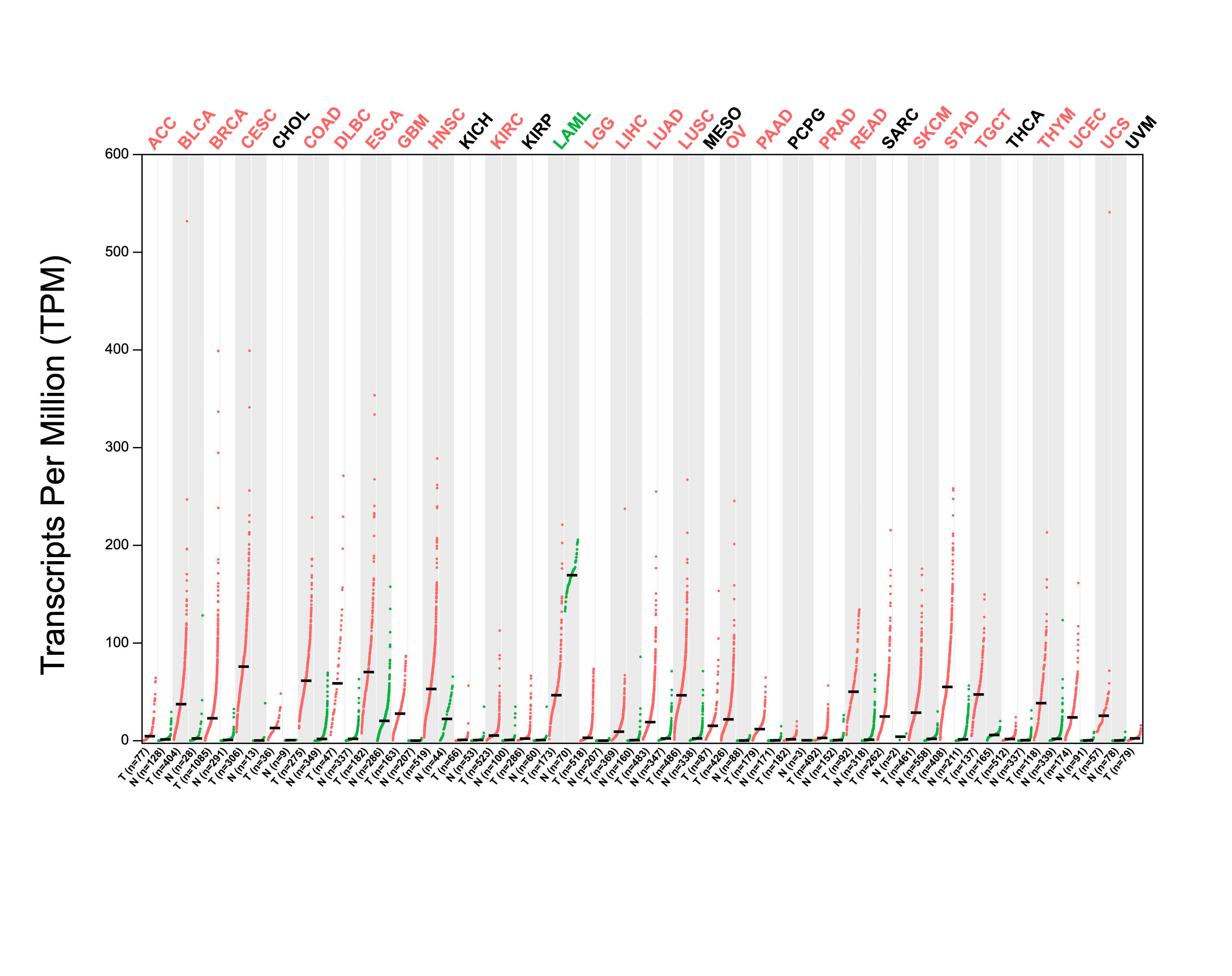

Supplement: Figure S1 [file peerj-10-14432-s008.png]

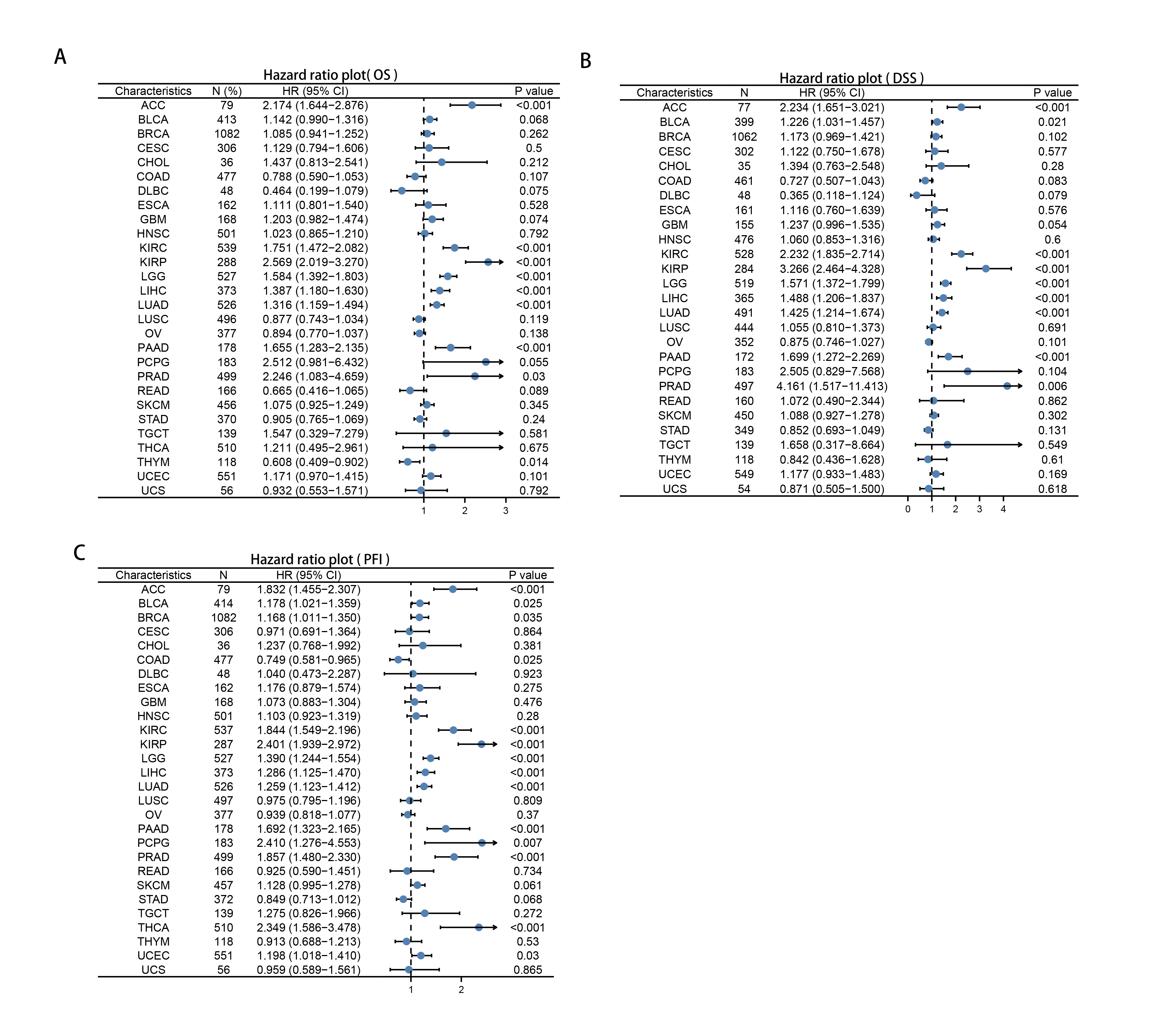

Supplement: Figure S2 — (A)OS, (B)DSS, (C)PFI. [file peerj-10-14432-s009.png]

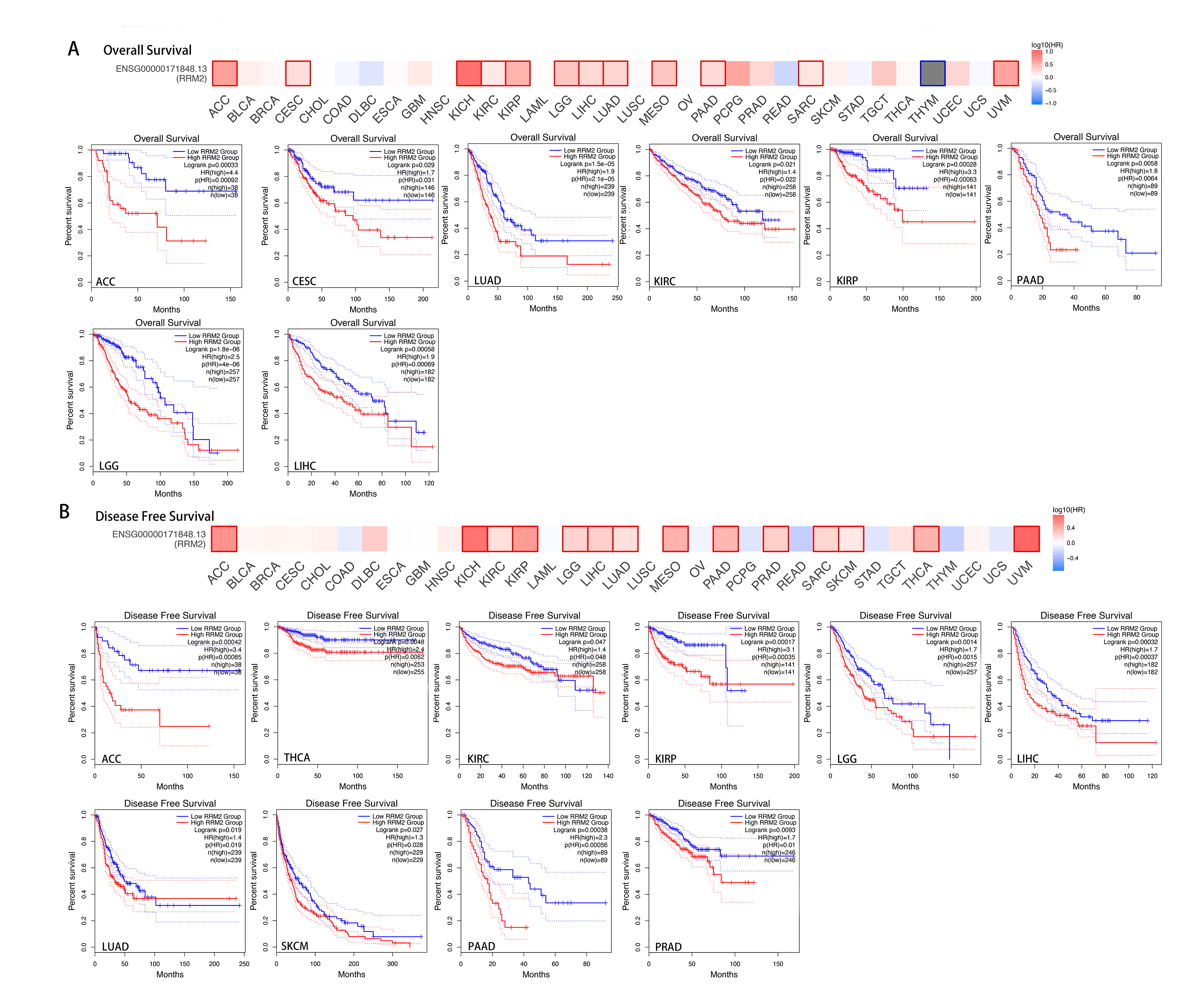

Supplement: Figure S3 — (A)OS, (B)DFS. The Kaplan-Meier curves with positive results are given. [file peerj-10-14432-s010.png]

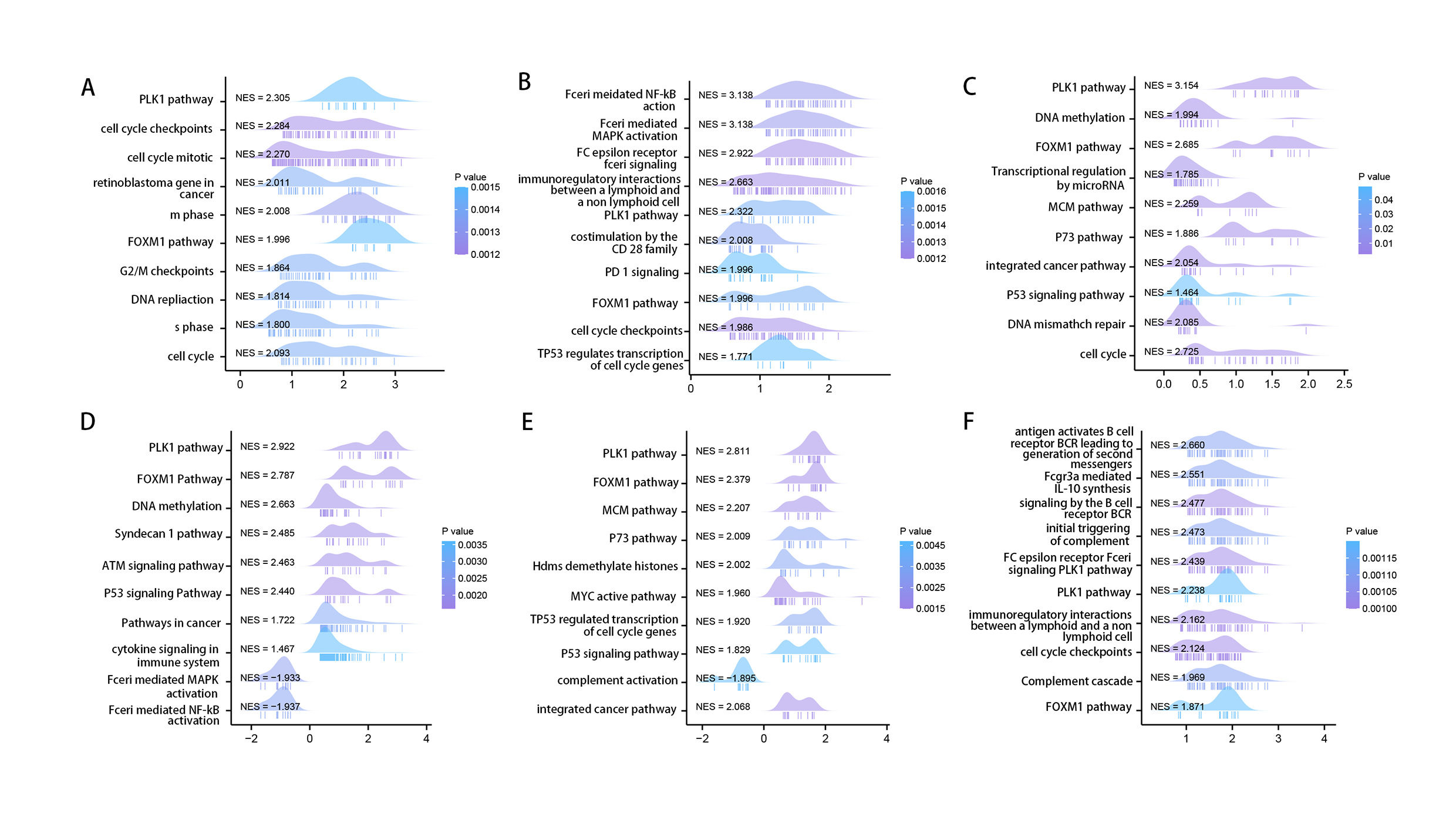

Supplement: Figure S4 — The 10 significant pathways of RRM2 GSEA results across the indicated tumor types. [file peerj-10-14432-s011.png]

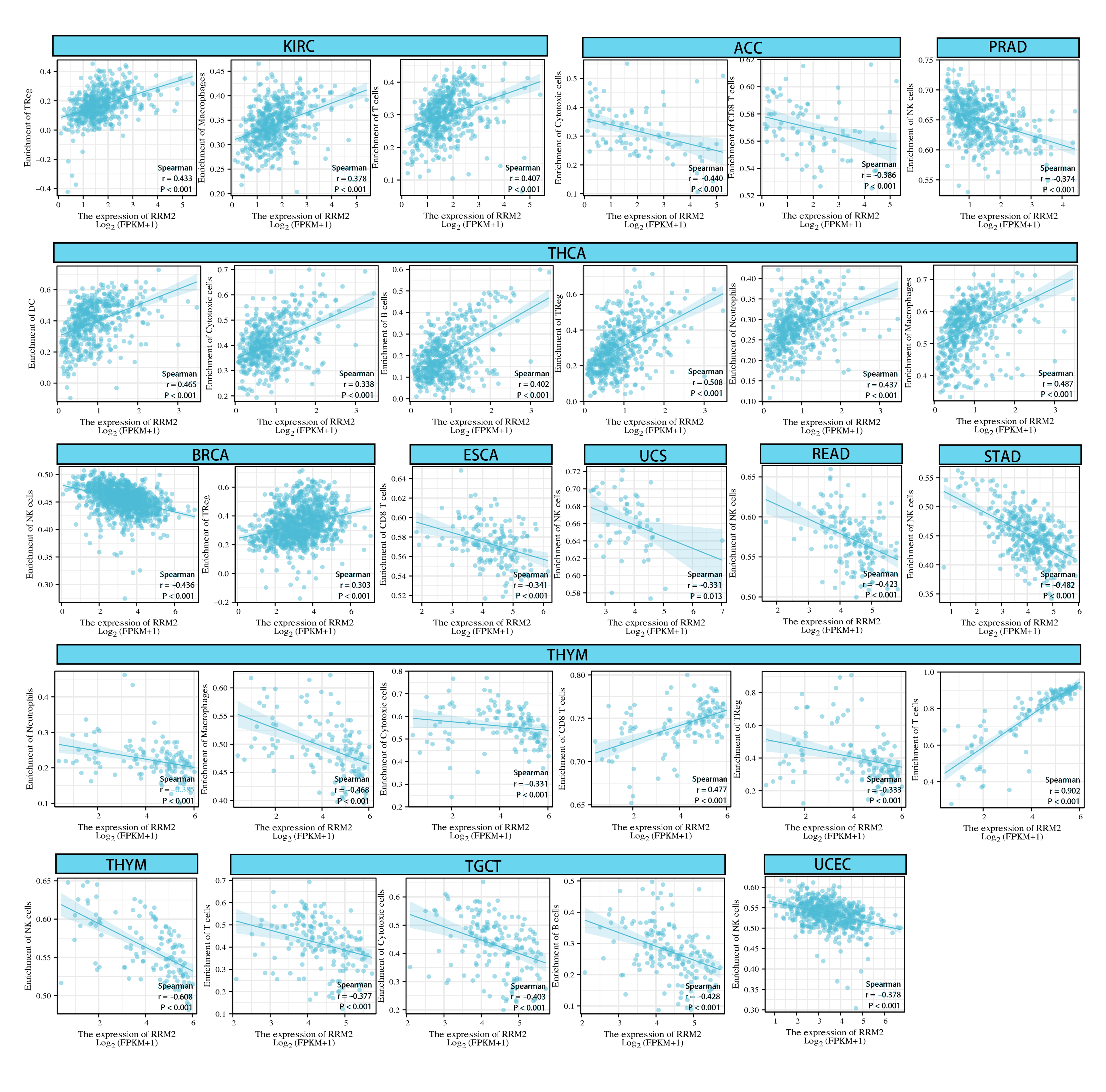

Supplement: Figure S5 [file peerj-10-14432-s012.png]
